# Supplementary figures and images for: A novel cascade allows Metarhizium robertsii to distinguish cuticle and hemocoel microenvironments during infection of insects
Source: PLoS Biol. 2021 Aug 4;19(8):e3001360. doi: 10.1371/journal.pbio.3001360 (PMC8366996; doi:10.1371/journal.pbio.3001360)

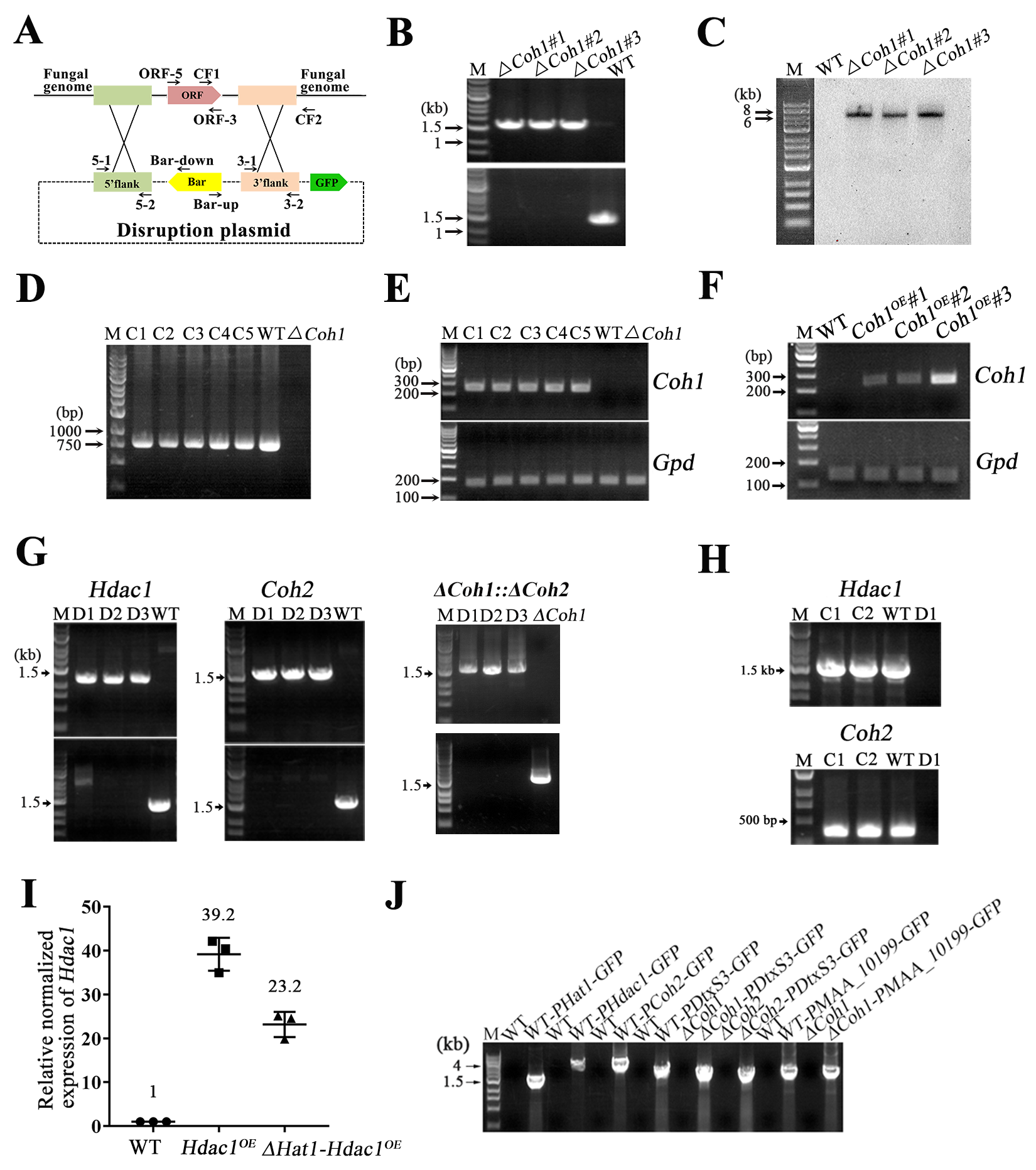

Supplement: S1 Fig — (A) The schematic diagram of gene deletion based on homologous recombination. Lower panel is a map of a deletion plasmid, and its relative position in the fungal genome is shown in the upper panel. (B) Confirmation of construction of the Coh1 deletion mutant (ΔCoh1) using PCR in the transformants with herbicide PPT resistance and without a GFP signal. ΔCoh1#1, ΔCoh1#2, and ΔCoh1#3 represent 3 independent isolates of the deletion mutant, and WT is the wild-type strain. Upper panel: PCR conducted with the primers Bar-up and the confirmation primer CF2 (the relative positions of all primers are shown in [A]). PCR products can be obtained only from the deletion mutants. Lower panel: PCR conducted with primers CF1 and CF2. PCR products can be obtained only in the WT strain. (C) Southern blot analysis confirms that the selection marker gene Bar was not ectopically integrated in the 3 isolates of the Coh1 deletion mutant shown in (B), indicating that the insertion of the selection marker gene only deleted the Coh1 gene. Genomic DNA (15 μg) was digested with BamHI and SmaI. The PCR product of the Bar gene was digoxigenin (DIG)–labeled and used as the DNA probe. (D) Confirmation of the insertion of the genomic clone of the Coh1 gene into the genome of the deletion mutant ΔCoh1 for the complementation by PCR using the primers ORF-5 and ORF-3. C1, C2, C3, C4, and C5 represent 5 randomly selected transformants. (E) Reverse transcription PCR (RT-PCR) analysis of Coh1 expression in the 5 randomly selected transformants (C1, C2, C3, C4, and C5) shown in (D), suggesting that construction of the complemented strain of ΔCoh1 failed due to the importance of the native chromosomal position of Coh1 for its transcriptional regulation. RNA was collected from the mycelium grown on PDA plates, where Coh1 was not expressed in the WT strain. Upper panel: the Coh1 gene. Note: No PCR product was seen in the WT strain and the deletion mutant ΔCoh1. Lower panel: the reference gene Gpd encoding gl [file pbio.3001360.s002.tif]

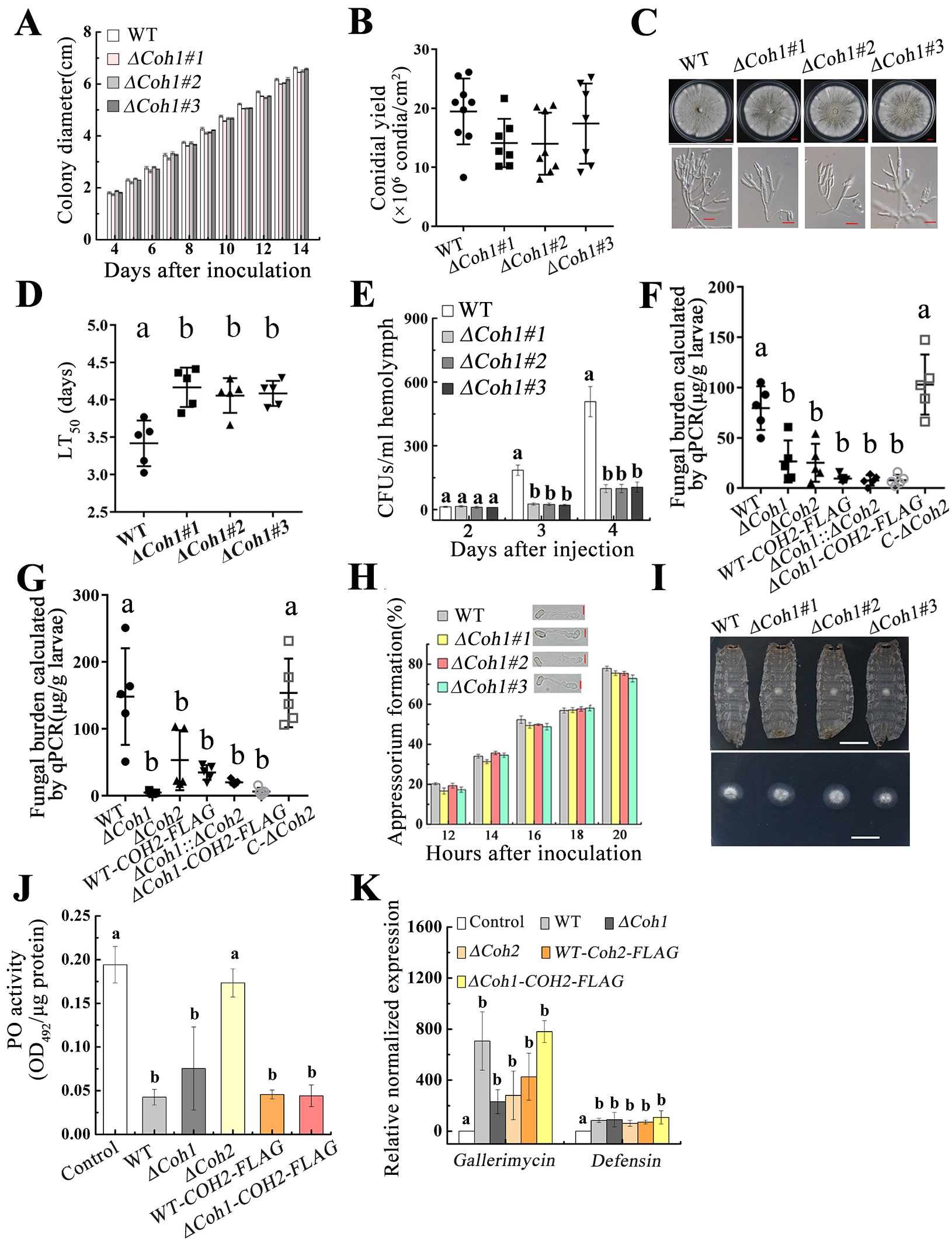

Supplement: S2 Fig — (A) The colony diameter on PDA plates. ΔCoh1#1, ΔCoh1#2, and ΔCoh1#3 are 3 independent isolates of the mutant ΔCoh1. Note: At each time point after inoculation, no significant difference in colony diameter was found between all tested strains (n = 3, P > 0.05, Tukey’s test in one-way ANOVA). (B) Conidial yields on PDA plates. Quantification of conidial yield was repeated 3 times with 3 replicates per repeat. Data are expressed as mean ± SE. No significant difference in conidial yield was found between the strains (n = 9, P > 0.05, one-way ANOVA). (C) Colony phenotypes (upper panel) and conidiophores (lower panel) on PDA plates of the 4 strains described in (A). Conidiophores were observed 5 d after inoculation (scale bar represents 10 μm), while colony picture was taken 18 d after inoculation (scale bar represents 10 mm). Images are representative of at least 3 independent experiments. (D) LT50 (time taken to kill 50% of insects) values when the insects were inoculated by injection of conidia into the hemocoel. Data are expressed as mean ± SE. Values with different letters are significantly different (n = 3, P < 0.05, Tukey’s test in one-way ANOVA). (E) Quantification of hyphal bodies in the insect hemocoel at day 2, 3, and 4 after injection of conidia into the hemocoel. CFU, colony forming units. Within each day, values with different letters are significantly different (n = 3, P < 0.05, Tukey’s test in one-way ANOVA). (F and G) Absolute qPCR analysis of fungal burden in live insects infected by M. robertsii strains via (F) topical application or (G) direct injection. Values with different letters are significantly different (n = 5, P < 0.05, Tukey’s test in one-way ANOVA). (H) Appressorial development on a hydrophobic plastic surface. For all time points, no significant difference in appressorial formation was found between the WT strain and the 3 independent isolates of the mutant ΔCoh1 (n = 3, P > 0.05, Tukey’s test in one-way ANOVA). The experiment was repeated [file pbio.3001360.s003.tif]

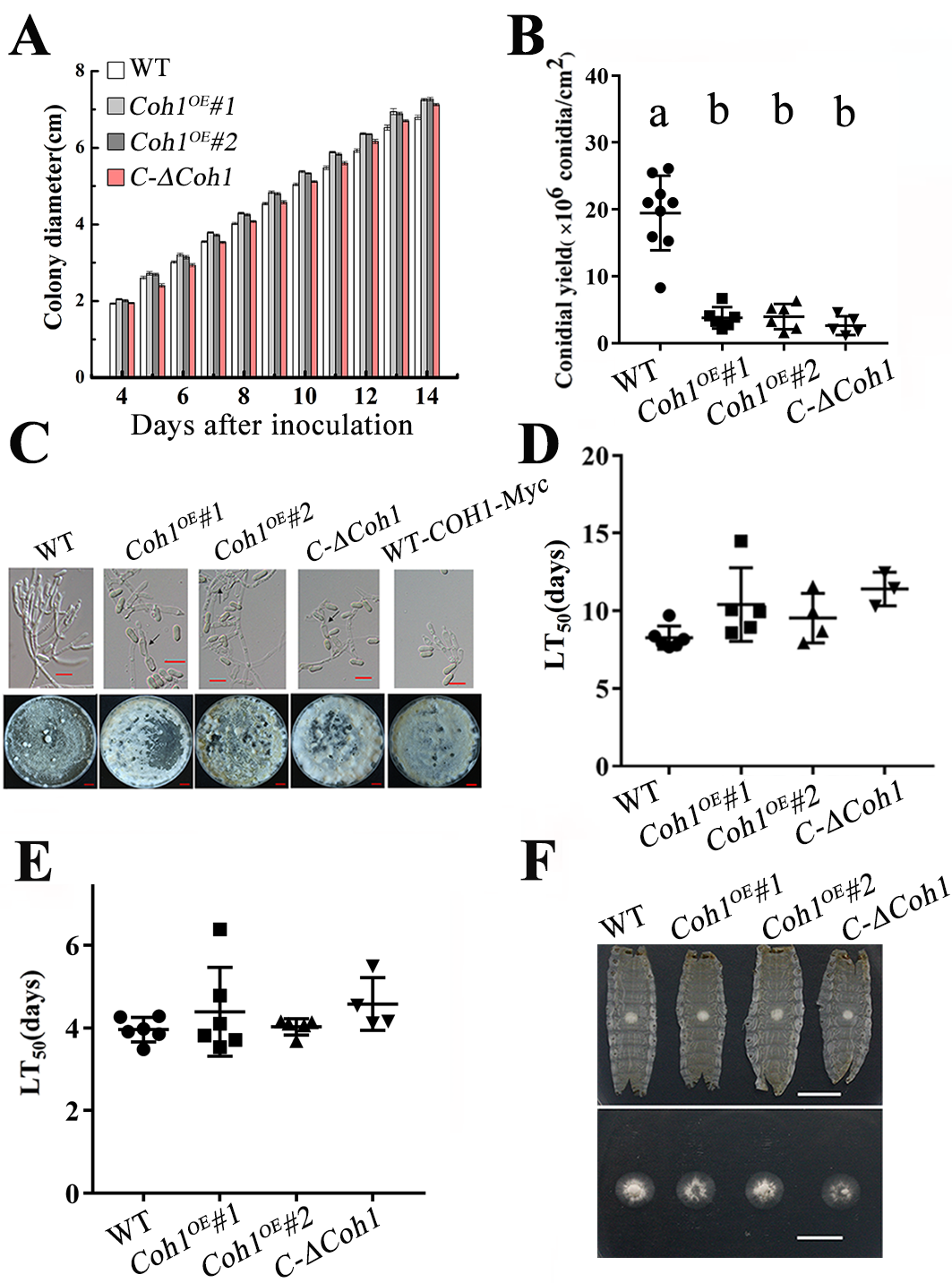

Supplement: S3 Fig — (A) Colony growth on PDA plates. Coh1OE#1 and Coh1OE#2 are 2 independent isolates of the strain Coh1OE. No significant difference was found between the strains at each time point (n = 9, P > 0.05, one-way ANOVA). (B) Conidial yield on PDA plates. Values with different letters are significantly different (n = 9, P < 0.05, Tukey’s test in one-way ANOVA). (C) Conidiophores (upper panel; scale bar: 10 μm) and colony morphology (lower panel; scale bar: 1 cm) on PDA plates. (D and E) LT50 values via (D) topical application or (E) direct injection. No significant difference was found between tested strains (P > 0.05, Tukey’s test in one-way ANOVA). (F) Cuticle penetration. The scale bar represents 1 cm. Images are representative of 3 independent experiments. The data underlying all the graphs shown in this figure can be found in S1 Data. (TIF) [file pbio.3001360.s004.tif]

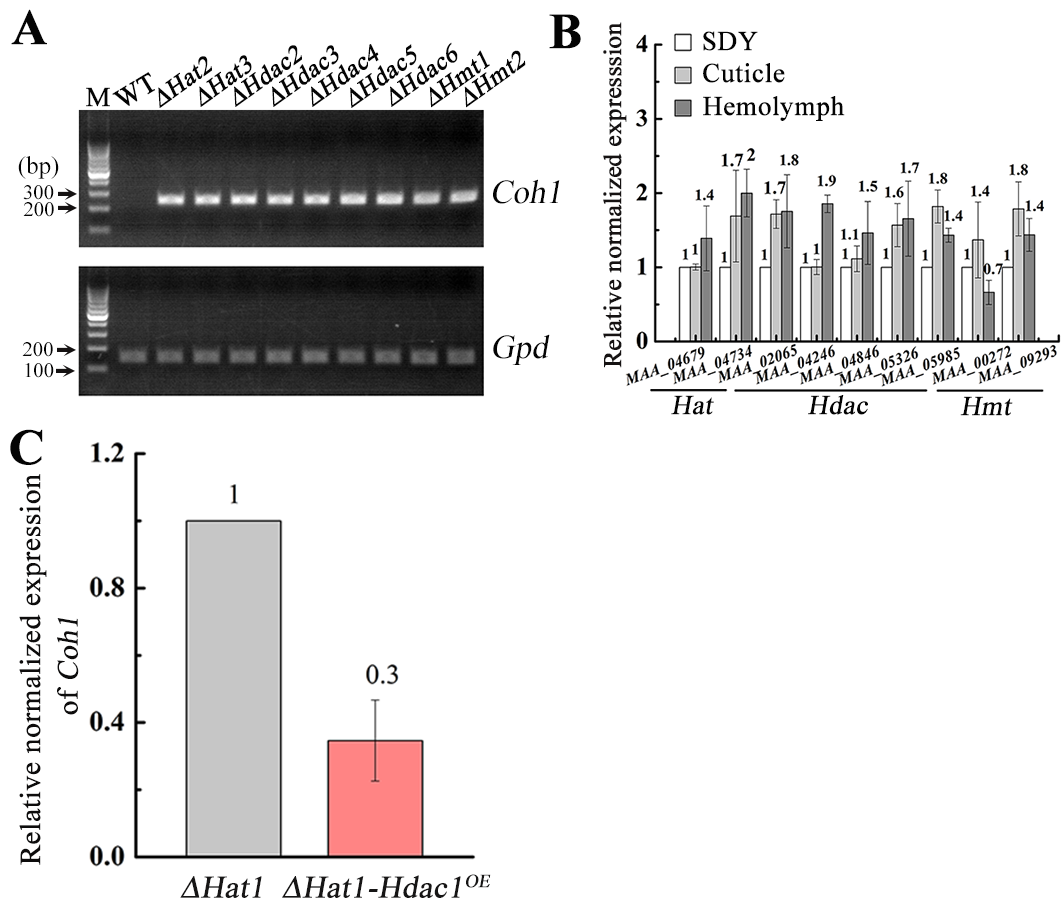

Supplement: S4 Fig — (A) Reverse transcription PCR (RT-PCR) analysis of Coh1 expression in the WT strain and the deletion mutants of 2 histone acetyltransferase genes (Hat2 [MAA_04679] and Hat3 MAA_04734]), 5 histone deacetylase genes (Hdac2 [MAA_02065], Hdac3 [MAA_04246], Hdac4 [MAA_04846], Hdac5 [MAA_05326], and Hdac6 [MAA_05985]), and 2 histone methyltransferase genes (Hmt1 [MAA_00272] and Hmt2 [MAA_09293]) during saprophytic growth. Note: No RT-PCR product was seen in the WT strain. Upper panel: the Coh1 gene; lower panel: the reference gene Gpd. M, DNA ladder. Images are representative of 3 independent experiments. (B) qRT-PCR analysis of the expression of the 9 epigenetic genes described in (A) during surrogate hemocoel colonization (Hemolymph) and cuticle penetration (Cuticle) relative to saprophytic growth (SDY). (C) qRT-PCR analysis of Coh1expression in the strains ΔHat1 and ΔHat1-Hdac1OE during surrogate hemocoel colonization. The data underlying all the graphs shown in this figure can be found in S1 Data. (TIF) [file pbio.3001360.s005.tif]

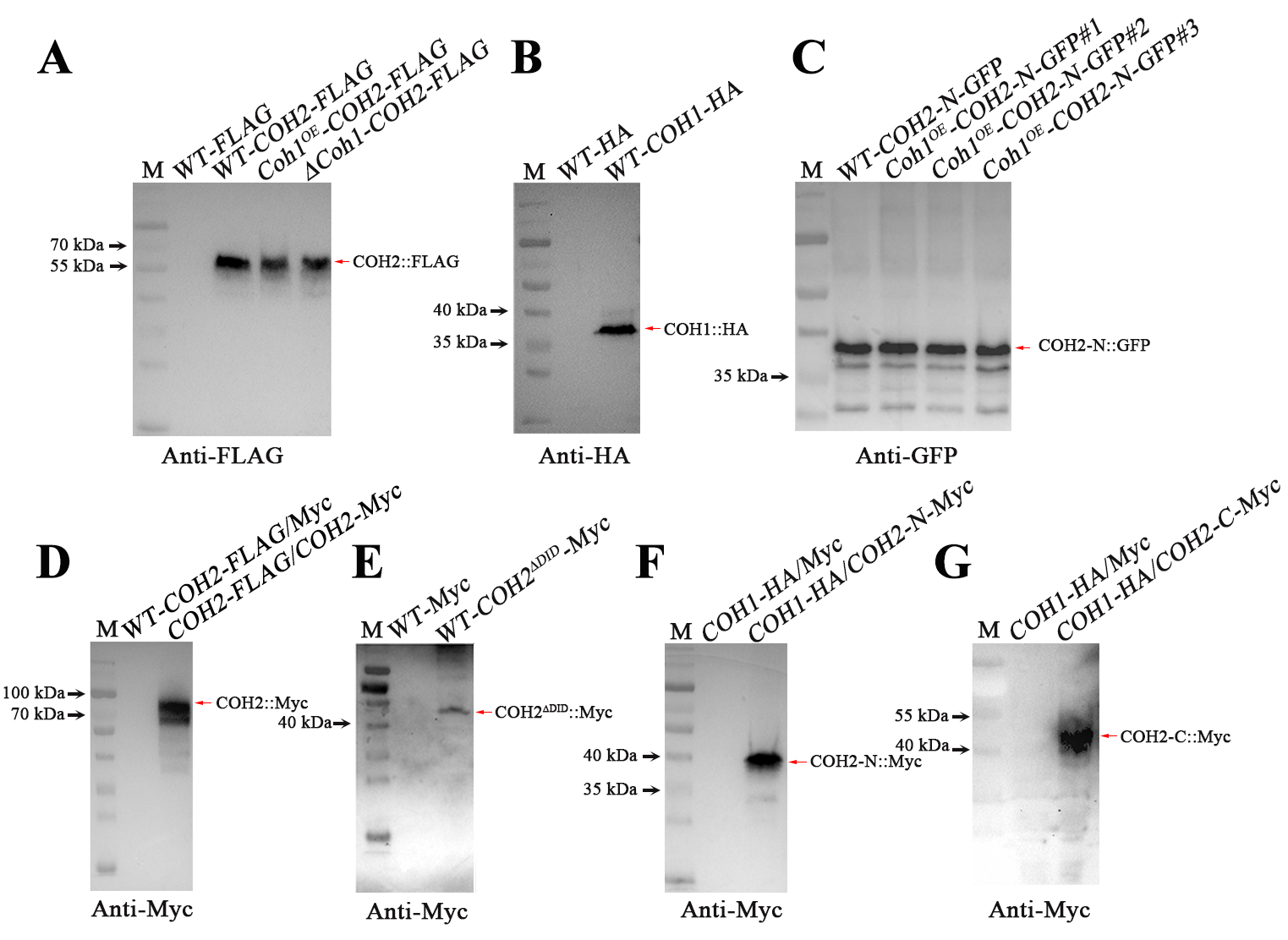

Supplement: S5 Fig — (A) Expression of the fusion protein COH2::FLAG in the WT strain to produce the strain WT-COH2-FLAG, in the strain Coh1OE to generate Coh1OE-COH2-FLAG, and in the mutant ΔCoh1 to form ΔCoh1-COH2-FLAG. The strain WT-FLAG was obtained by transforming the WT strain with the plasmid pPK2-Bar-Ptef-FLAG for the expression of the FLAG tag. Note: Due to dimerization, the band corresponding to the fusion protein COH2::FLAG was approximately 2-fold bigger than its predicted molecular weight. The band corresponding to the FLAG tag was not seen due to its small size. (B) Expression of the fusion protein COH1::HA in the WT strain to produce the strain WT-COH1-HA. The strain WT-HA was the WT strain transformed with the plasmid pPK2-Sur-Ptef-HA for expressing the HA tag. (C) Expression of the fusion protein COH2-N::GFP with the N-terminus (Met-1 to Thr-100) of COH2 tagged with the GFP protein in the WT strain to produce the strain WT-COH2-N-GFP and in the strain Coh1OE to produce Coh1OE-COH2-N-GFP. Coh1OE-COH2-N-GFP#1, Coh1OE-COH2-N-GFP#2, and Coh1OE-COH2-N-GFP#3 are 3 independent isolates of the strain Coh1OE-COH2-N-GFP. (D) Expression of the fusion protein COH2::Myc in the strain WT-COH2-FLAG to produce the strain COH2-FLAG/COH2-Myc. The strain WT-COH2-FLAG/Myc is the strain WT-COH2-FLAG transformed with the plasmid pPK2-Bar-Ptef-Myc for expressing the Myc tag. (E) Expression of the protein with the tag Myc fused to the mutated COH2 (COH2ΔDID, substituting the 7 leucine residues in the dimer interface domain with alanine) in the WT strain to produce the strain WT-COH2ΔDID-Myc. The strain WT-Myc is the WT strain transformed with the plasmid pPK2-Bar-Ptef-Myc for expressing the Myc tag. (F) Expression of the fusion protein COH1::HA and the protein with the N-terminus of COH2 fused to the Myc tag in the WT strain to produce the strain COH1-HA/COH2-N-Myc. The strain COH1-HA/Myc is obtained by transforming the plasmid pPK2-Bar-Ptef-Myc into the strain WT-COH1-HA. (G) Expression of th [file pbio.3001360.s006.tif]

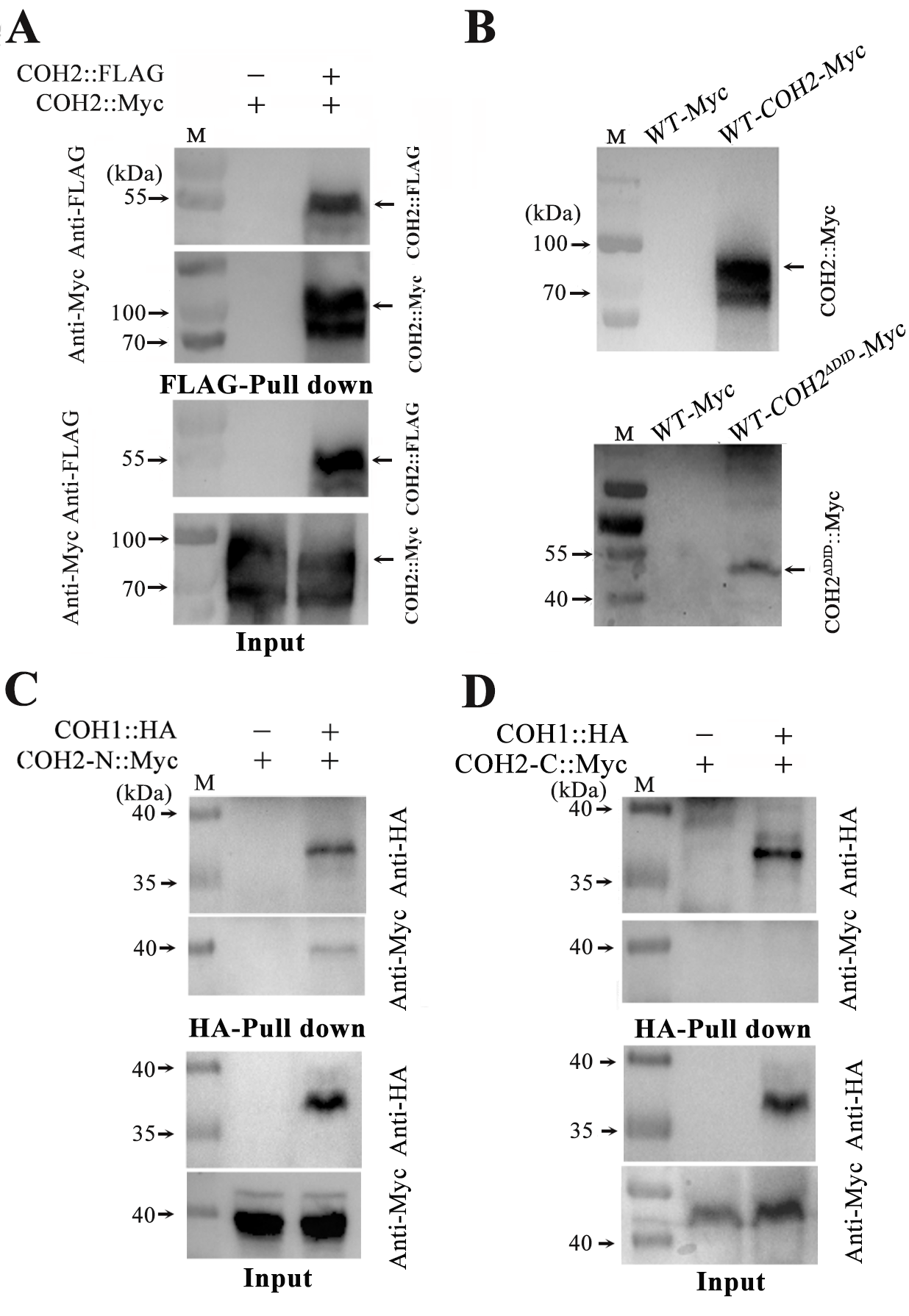

Supplement: S6 Fig — (A) Co-IP confirmation of the formation of COH2 dimer. The fusion proteins COH2::FLAG (molecular weight = 33 kDa) and COH2::Myc (molecular weight = 44.6 kDa) were simultaneously expressed in the strain COH2-FLAG/COH2-Myc. The control strain COH2-Myc expressed the protein COH2::Myc. Immunoprecipitation was conducted with an anti-FLAG antibody. Proteins were detected by immunoblot analysis with anti-Myc or anti-FLAG antibodies. Upper panel: arrow indicates the dimer of COH2::FLAG; lower panel: arrow indicates the dimer of COH2::Myc and COH2::FLAG. (B) The leucine residues in the dimer interface domain (DID) are key to COH2 dimerization. WT-COH2-Myc: a strain expressing the protein COH2::Myc; WT-COH2ΔDID-Myc: a strain expressing the Myc-tagged protein COH2ΔDID, a mutated COH2 with the 7 leucine residues in the DID substituted to alanine. Note: The protein COH2::Myc forms a dimer (upper panel), whereas COH2ΔDID::Myc does not (lower panel). (C) Co-IP analysis shows that the Myc-tagged N-terminus of COH2 (COH2-N::Myc) physically interacts with the HA-tagged COH1 (COH1::HA). (D) Co-IP analysis shows that the Myc-tagged C-terminus of COH2 (COH2-C::Myc) does not interact with the HA-tagged COH1 (COH1::HA). Immunoprecipitation was conducted with anti-HA antibody. Proteins were detected by immunoblot analysis with anti-HA or anti-Myc antibodies. (TIF) [file pbio.3001360.s007.tif]

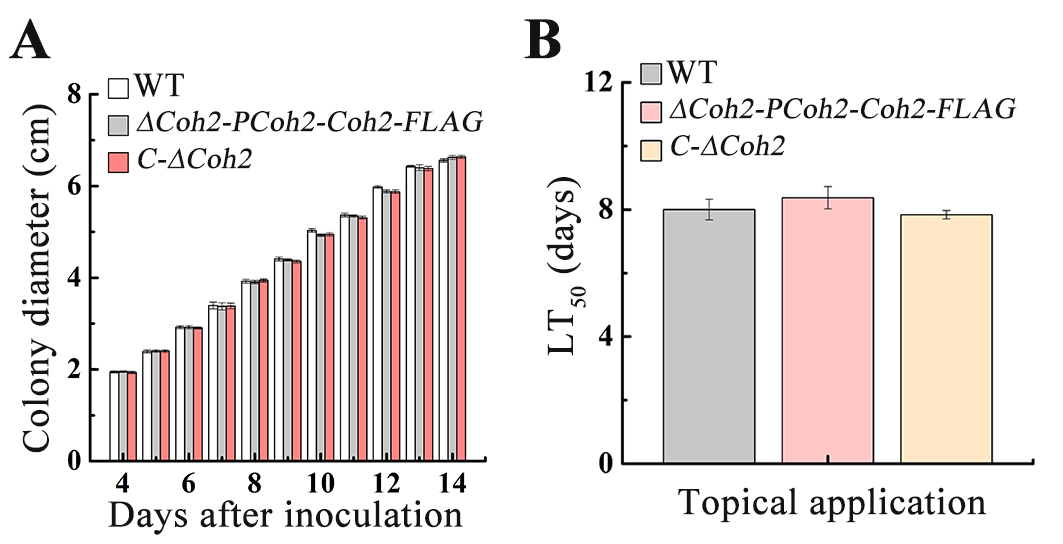

Supplement: S7 Fig — (A) Colony diameters of the WT, ΔCoh2-PCoh2-COH2-FLAG, and C-ΔCoh2 strains. Note: There was no significant difference between the 3 strains within each day (n = 9, P > 0.05, Tukey’s test in one-way ANOVA). (B) LT50 values via topical application. No significant difference was found between the tested strains (n = 3, P > 0.05, Tukey’s test in one-way ANOVA). The data underlying all the graphs shown in this figure can be found in S1 Data. (TIF) [file pbio.3001360.s008.tif]

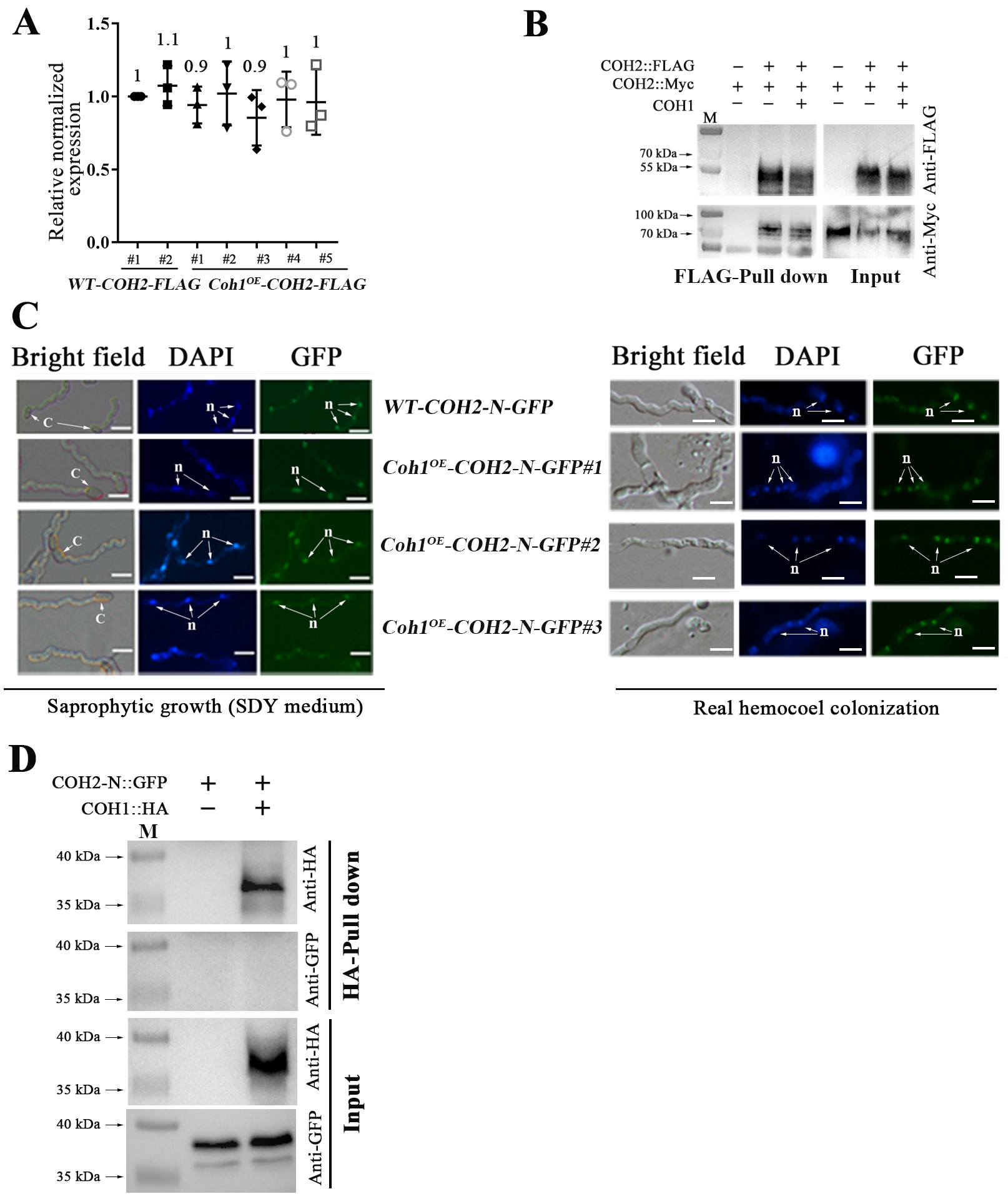

Supplement: S8 Fig — (A) qRT-PCR analysis of the expression of the gene encoding the fusion protein COH2::FLAG in 2 isolates of the strain WT-COH2-FLAG and 5 isolates of the strain Coh1OE-COH2-FLAG. (B) Co-IP analysis shows that the COH2 protein dimerized in the Coh1-overexpressing strain Coh1OE. Three strains were used: (1) WT-COH2-Myc, constitutively expressing the fusion protein COH2::Myc; (2) WT-COH2-FLAG/COH2-Myc, constitutively expressing the fusion proteins COH2::FLAG and COH2::Myc; and (3) Coh1OE-COH2-FLAG/COH2-Myc, which constitutively expressed the COH1 protein and the fusion proteins COH2::FLAG (molecular weight = 33 kDa) and COH2::Myc (molecular weight = 44.6 kDa). The strains were grown in the nutrient-rich medium SDY, where COH1 was not expressed in the strain WT-COH2-FLAG/COH2-Myc. Immunoprecipitations were conducted with an anti-FLAG antibody. Proteins were detected by immunoblot analysis with anti-Myc or anti-FLAG antibodies. Note: The fusion proteins COH2::FLAG and COH2::Myc dimerized in the strains Coh1OE-COH2-FLAG/COH2-Myc and WT-COH2-FLAG/COH2-Myc, and the fusion protein COH2::Myc formed a dimer in the strain WT-COH2-Myc. (C) GFP-tagged N-terminus of COH2 enters into the nucleus during saprophytic growth in SDY medium (left panel) and real hemocoel colonization (right panel). Two strains were used: (1) WT-COH2-N-GFP, with the fusion protein COH2-N::GFP (GFP fused to the COH2 N-terminus) expressed in the WT strain, and (2) Coh1OE-COH2-N-GFP, constitutively expressing the COH1 protein and COH2-N::GFP. Three independent isolates of the strain Coh1OE-COH2-N-GFP were randomly selected for this assay. In each panel: left, bright field microscopy; middle, fluorescence microscopy for DAPI (4′,6-diamidino-2-phenylindole) staining; right, fluorescence microscopy for GFP observation. C, conidium; N, nucleus. Note: In all tested strains, the GFP fluorescence intensity was strongest in the nucleus. (D) Co-IP analysis shows that the GFP-tagged N-terminus of COH2 (COH2-N::GFP) cou [file pbio.3001360.s009.tif]

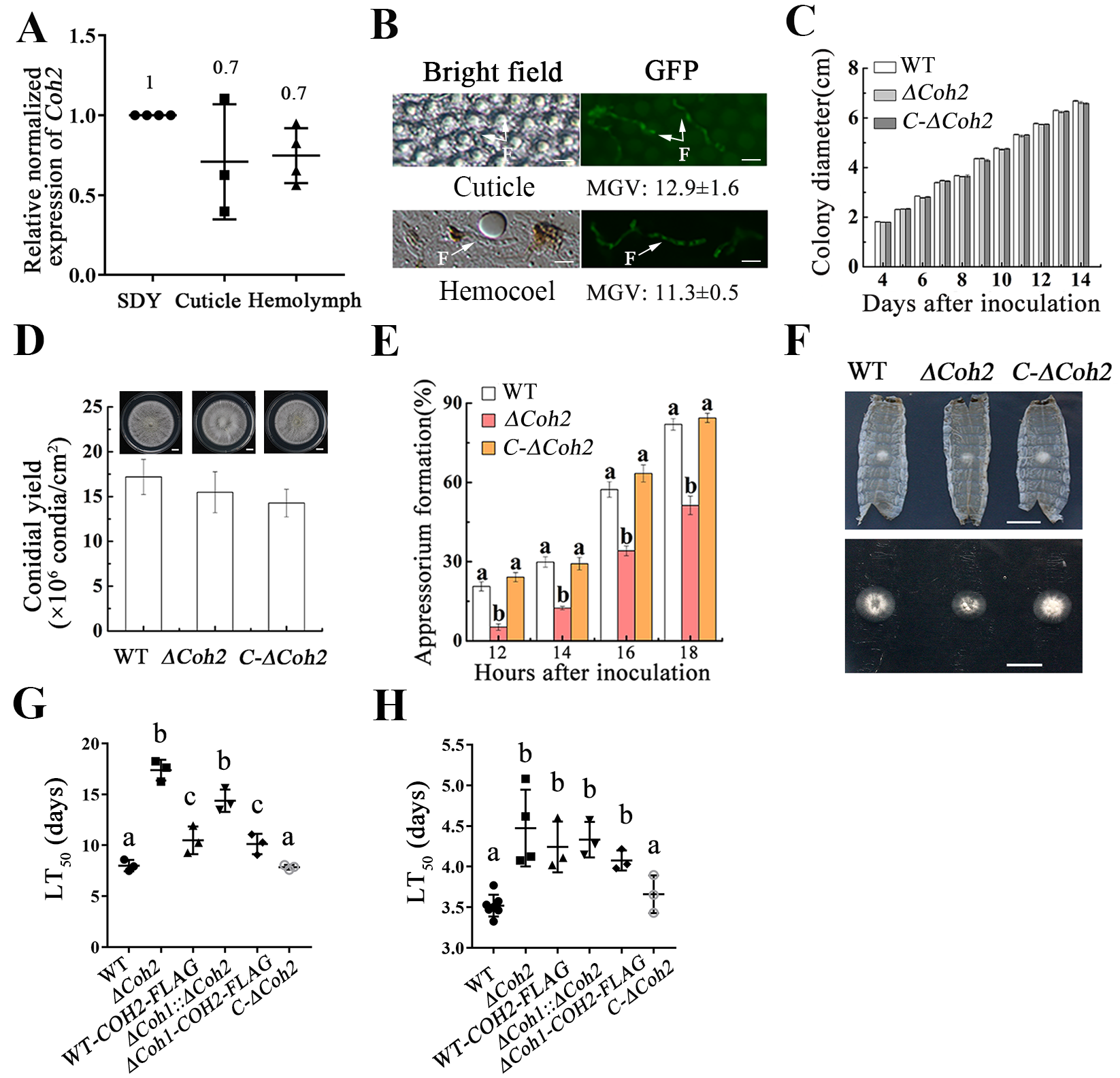

Supplement: S9 Fig — (A) qRT-PCR analysis of Coh2 expression during surrogate hemocoel colonization (Hemolymph), cuticle penetration (Cuticle), and saprophytic growth (SDY). (B) GFP signal in fungal cells of the strain with gfp driven by Coh2 promoter on the cuticle (Cuticle) and in the real hemocoel (Hemocoel) of G. mellonella larvae. F, fungal cells. Scale bar: 10 μm. Images are representative of 3 independent experiments. (C) Colony diameter on PDA plates of the WT strain, the mutant ΔCoh2, and its complemented strain C-ΔCoh2. Inoculations were conducted by applying 5 μL of a conidial suspension (1 × 10^7^ conidia/mL) on the center of a PDA plate. Note: Within each day, no significant difference in colony diameter was observed between the 3 strains. (D) Conidial yields. Note: No significant difference was seen between the 3 strains. The insets are the colony pictures that were taken at day 18 after inoculation on PDA plates (scale bar represents 10 mm). (E) Appressorial development on a hydrophobic plastic surface. At each time point, values with different letters are significantly different (n = 3, P < 0.05, Tukey’s test in one-way ANOVA). (F) Cuticle penetration. This experiment is described in S2I Fig. The scale bar represents 1 cm. Images are representative of 3 independent experiments. (G and H) LT50 values via (G) topical application or (H) injection. Data are expressed as mean ± SE. Values with different letters are significantly different (P < 0.05, Tukey’s test in one-way ANOVA). The data underlying all the graphs shown in this figure can be found in S1 Data. (TIF) [file pbio.3001360.s010.tif]

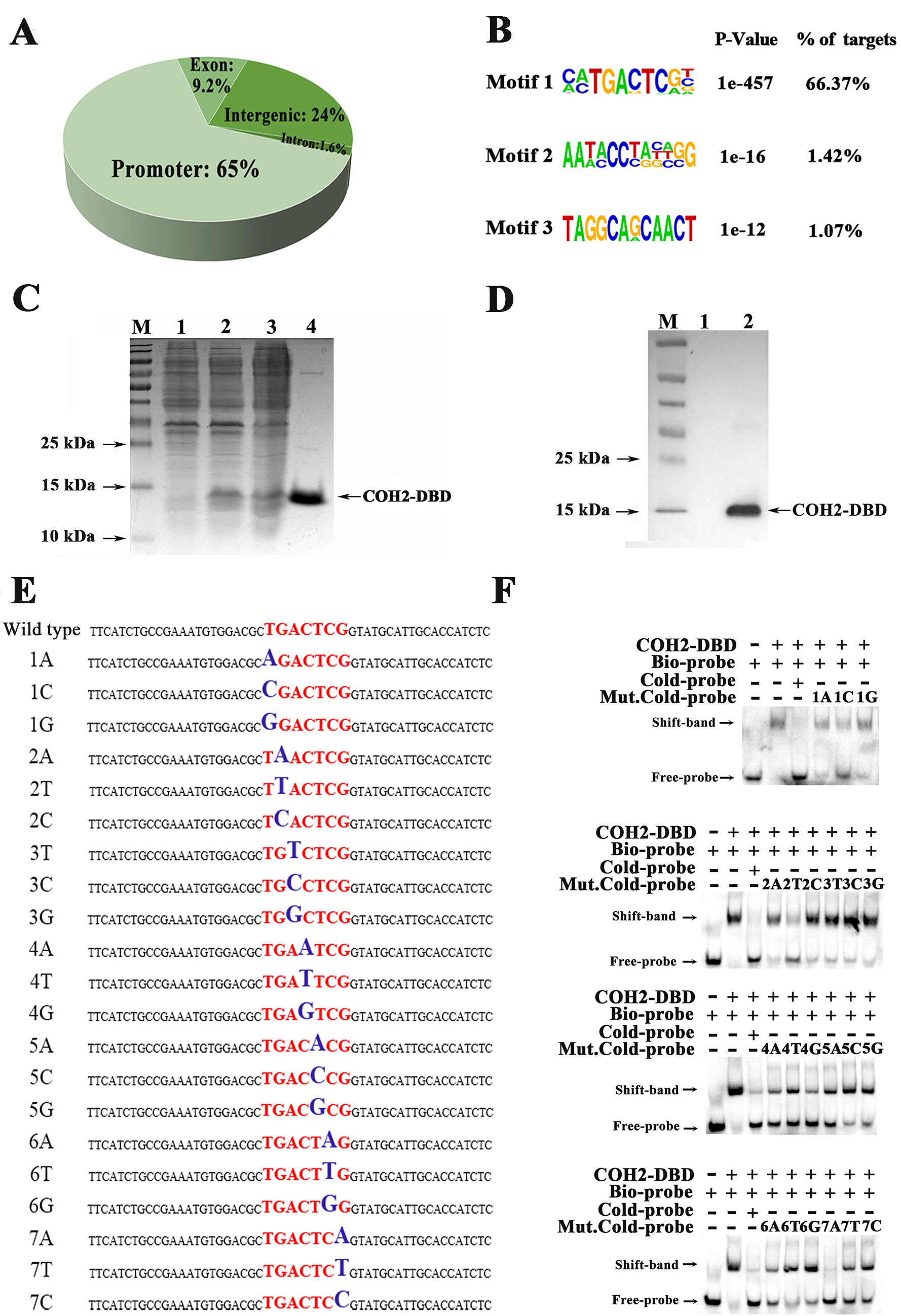

Supplement: S10 Fig — (A) Distribution of all COH2 peak locations within the genome revealed by ChIP-Seq analysis. (B) The top 3 binding motifs enriched by the HOMER (Hypergeometric Optimization of Motif EnRichment) software. (C) SDS-PAGE analysis of the expression and purification of the recombinant COH2-DBD (DNA binding domain of COH2) protein in E. coli. M, protein ladder (Thermo Fisher Scientific). Lane 1: Crude extract from the E. coli cells with the empty plasmid pET-28a (control). Lane 2: Crude extract from the E. coli cells expressing COH2-DBD. Lane 3: The supernatant of the crude extract shown in lane 2. Lane 4: Proteins purified from the supernatant shown in lane 3 with the HisPur Ni-NTA Resin. (D) Western blot analysis to confirm the expression of the recombinant COH2-DBD using the anti-His tag antibody. M, protein ladder. Lanes 1 and 2 are the proteins shown in lanes 1 and 4, respectively, in (C). (E) The sequences of the DNA probes containing the motif COH2-BM (shown in red), which is flanked by the sequences (black) in the promoter of the gene MAA_04430. The name of the DNA probe is shown on the left, and its sequence is on the right. Wild type: the DNA probe containing the wild-type motif COH2-BM; 1A: the nucleotide at position 1 in the motif COH2-BM is changed from the wild-type one (T) into the nucleotide A. The naming system is also used for all other mutated DNA probes shown in this figure. (F) EMSA analysis of the binding of the DNA probes to the recombinant COH2-DBD protein. The binding activity was demonstrated by the DNA (Bio-probe: the biotin-labeled wild-type DNA probe) band shift prior to the addition of the specific competitor (Cold probe: the unlabeled wild-type DNA probe) in a 300-fold excess. The importance of each nucleotide in the motif COH2-BM in its binding to the recombinant COH2-DBD protein is shown by the impact on the DNA (Bio-probe) band shift of adding an unlabeled mutated probe (Mut.Cold-probe) as a competitor in a 300-fold excess. The names of th [file pbio.3001360.s011.tif]

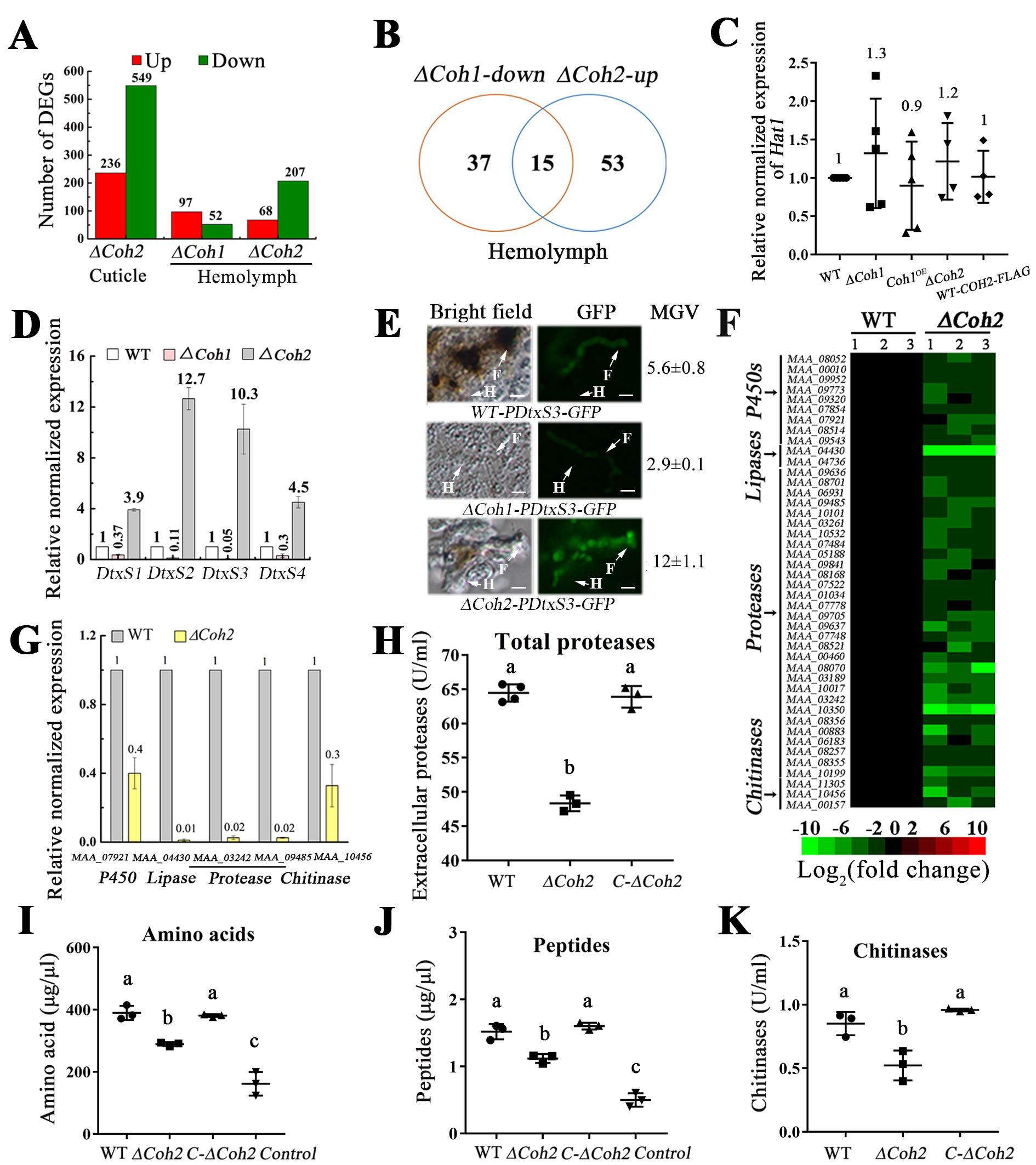

Supplement: S11 Fig — (A) A summary of differentially expressed genes profiled by RNA-Seq analysis between the WT strain and the mutants ΔCoh1 and ΔCoh2 during surrogate hemocoel colonization (Hemolymph) or cuticle penetration (Cuticle). Up (red) and down (green) represent the genes up-regulated and down-regulated in the mutants compared with the WT strain. (B) Venn diagram showing the distribution of genes positively regulated by COH1 and negatively regulated by COH2 during surrogate hemocoel colonization. (C) qRT-PCR analysis of Hat1 expression during surrogate hemocoel colonization in the WT strain, the mutants ΔCoh1 and ΔCoh2, the Coh1-overexpressing strain Coh1OE, and the Coh2-overexpressing strain WT-COH2-FLAG. (D) qRT-PCR analysis of expression of the 4 destruxin biosynthesis genes (DtxS1, DtxS2, DtxS3, and DtxS4) during surrogate hemocoel colonization in the WT strain and the mutants ΔCoh1 and ΔCoh2. (E) GFP signal in the fungal cells collected from the real hemocoel of insects infected by the strains WT-PDtxS3-GFP, ΔCoh1-PDtxS3-GFP, and ΔCoh2-PDtxS3-GFP. In the 3 strains, the gfp gene was driven by the DtxS3 promoter. F, fungal cells; H, hemocyte; MGV, mean gray value. Scale bar represents 10 μm. (F) RNA-Seq analysis of regulation of the chitinase, protease, lipase, and P450 genes by COH2 during cuticle penetration. The expression level of a gene in the WT strain is set to 1; the values represent the log2-transformed fold-changes of differential gene expression in the mutant ΔCoh2. The 3 individual experiments are indicated by the numbers 1, 2, and 3, respectively. (G) qRT-PCR analysis of expression of cuticle-degrading genes during cuticle penetration in the mutant ΔCoh2 and the WT strain. For quantification of free amino acids, peptides, and activities of cuticle-degrading enzymes, the fungal strains were grown in cuticle medium using G. mellonella cuticle as the sole carbon and nitrogen source. (H) Total extracellular protease activity in culture supernatants. (I and J) The c [file pbio.3001360.s012.tif]
