# Supplementary material for: A novel cascade allows Metarhizium robertsii to distinguish cuticle and hemocoel microenvironments during infection of insects
Source: PLoS Biol. 2021 Aug 4;19(8):e3001360. doi: 10.1371/journal.pbio.3001360 (PMC8366996; doi:10.1371/journal.pbio.3001360)

Fig. 1A

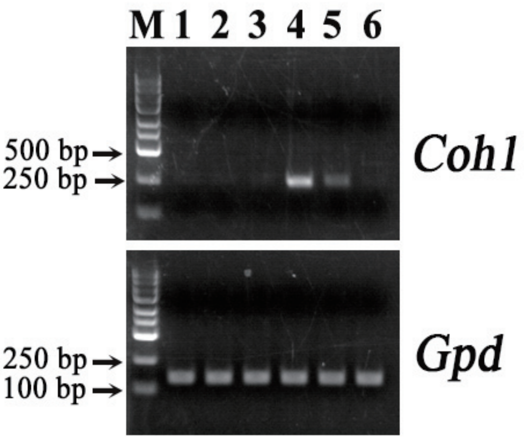

Fig. 1C

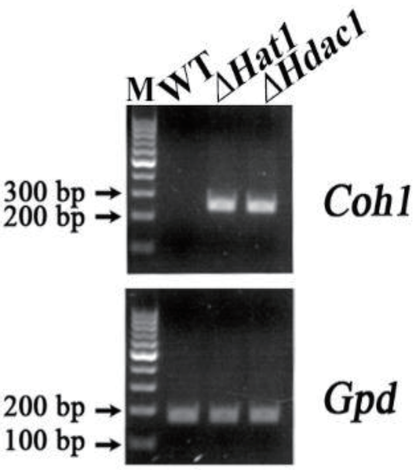

Fig. 1E

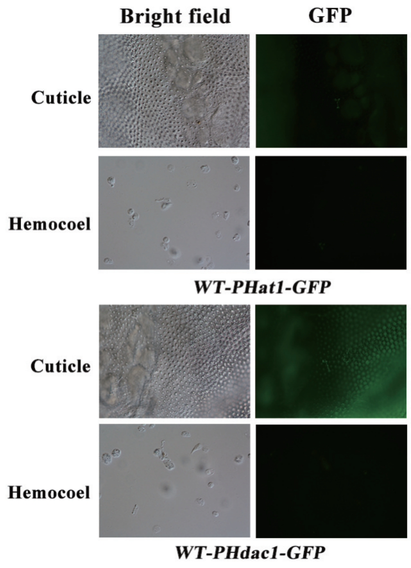

Fig. 2A

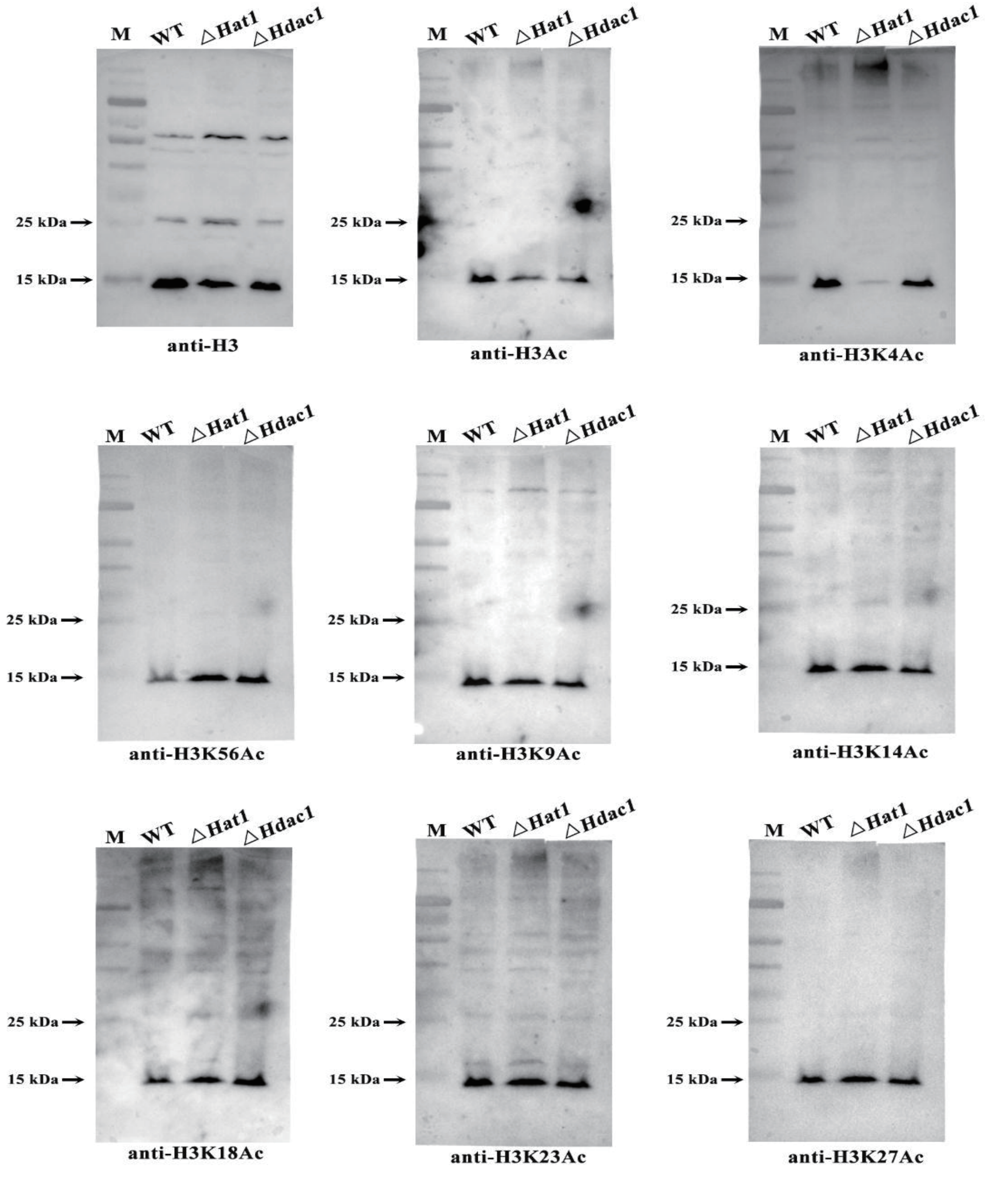

Fig. 3B

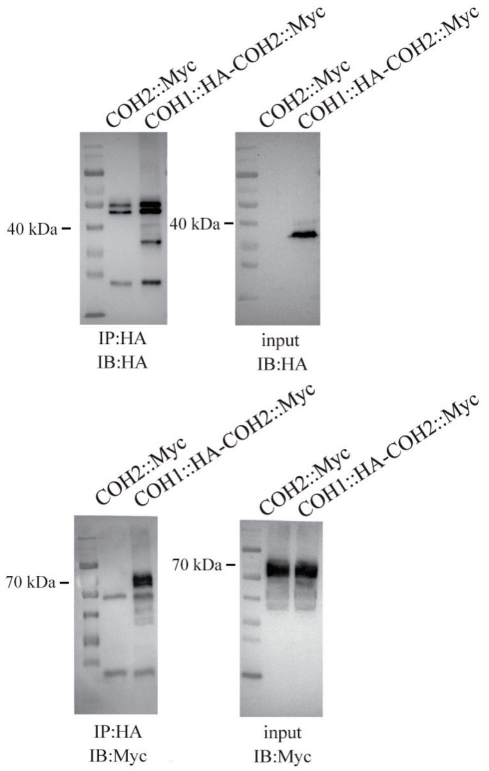

Fig. 3C

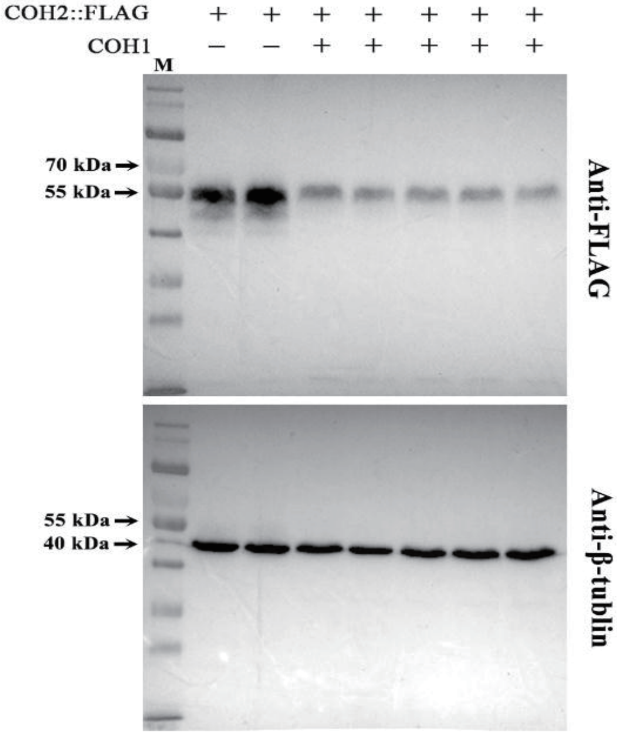

Fig. 3D

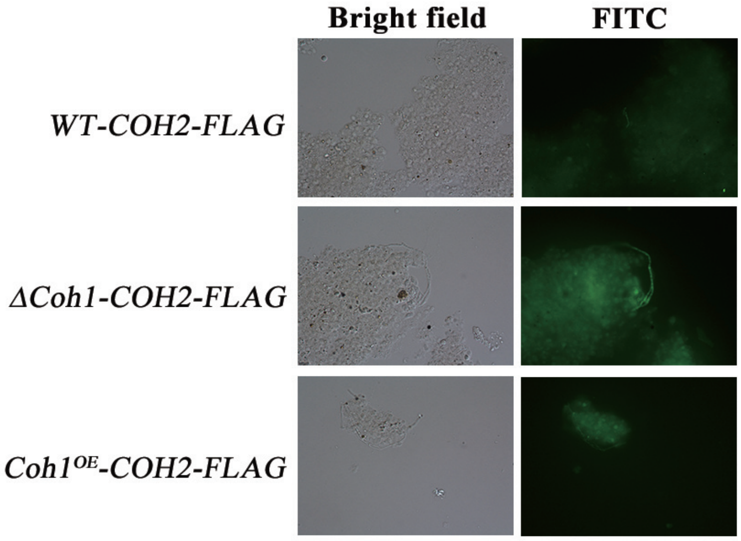

Fig. 3E

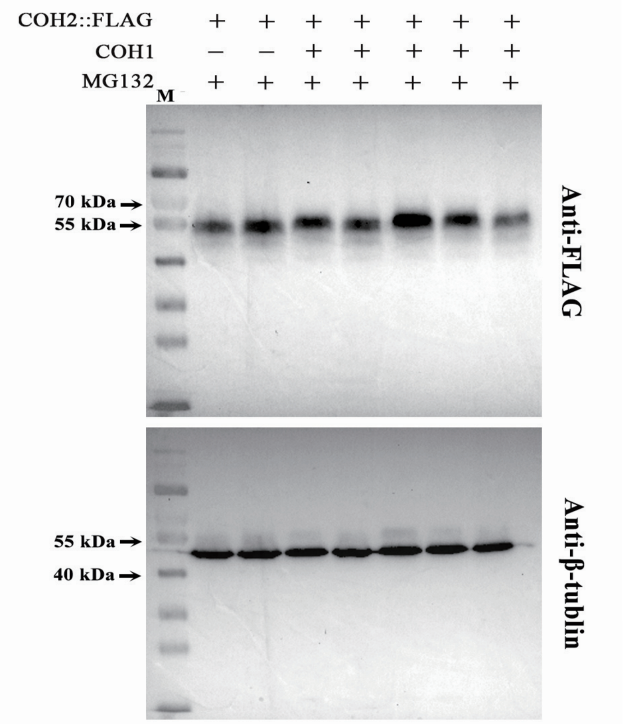

Fig. 3F

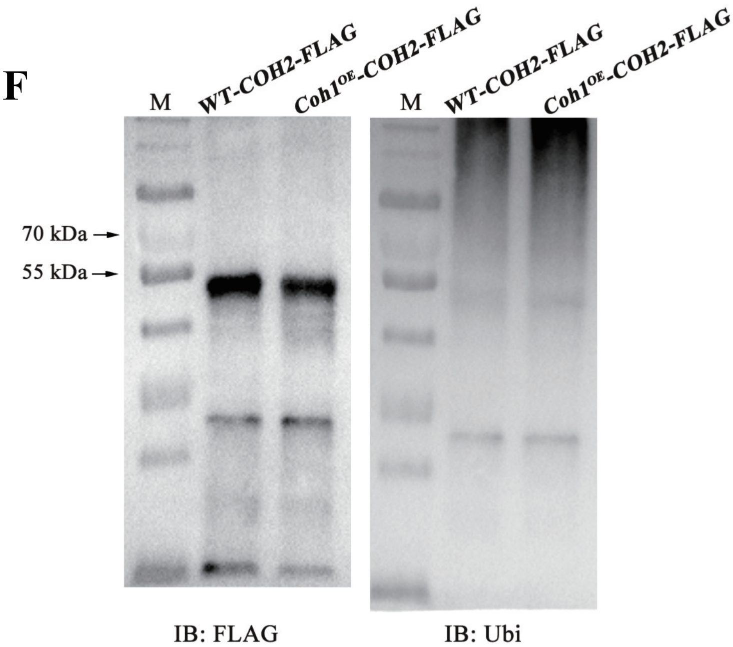

Fig. 4B

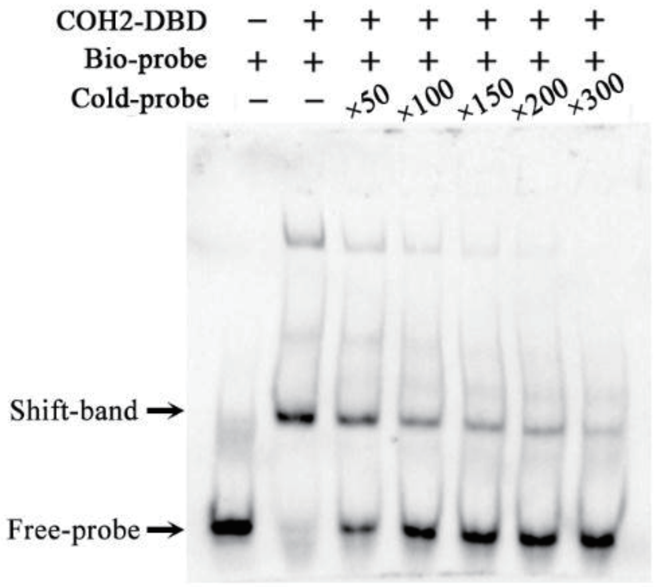

Fig. 5G

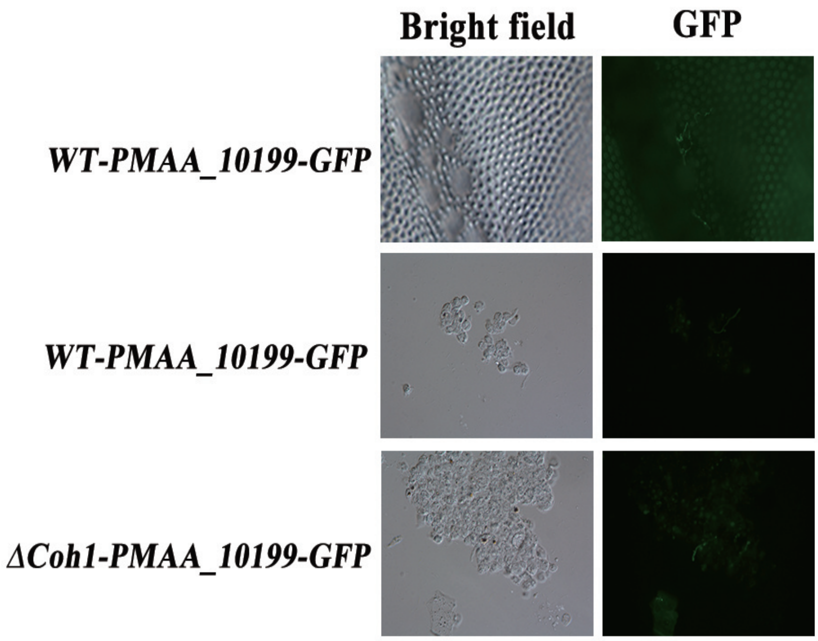

Fig. S1

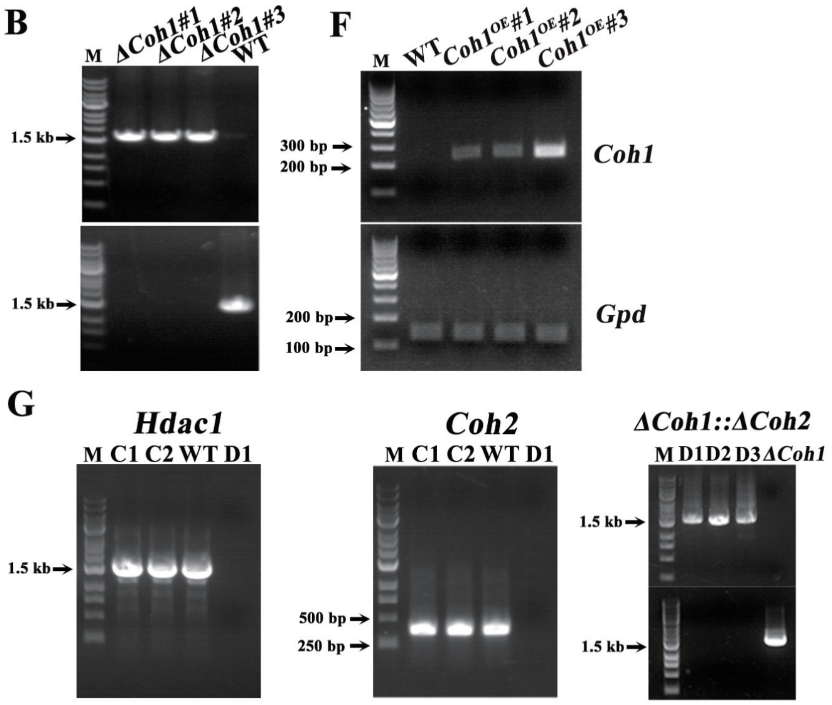

Fig. S6A

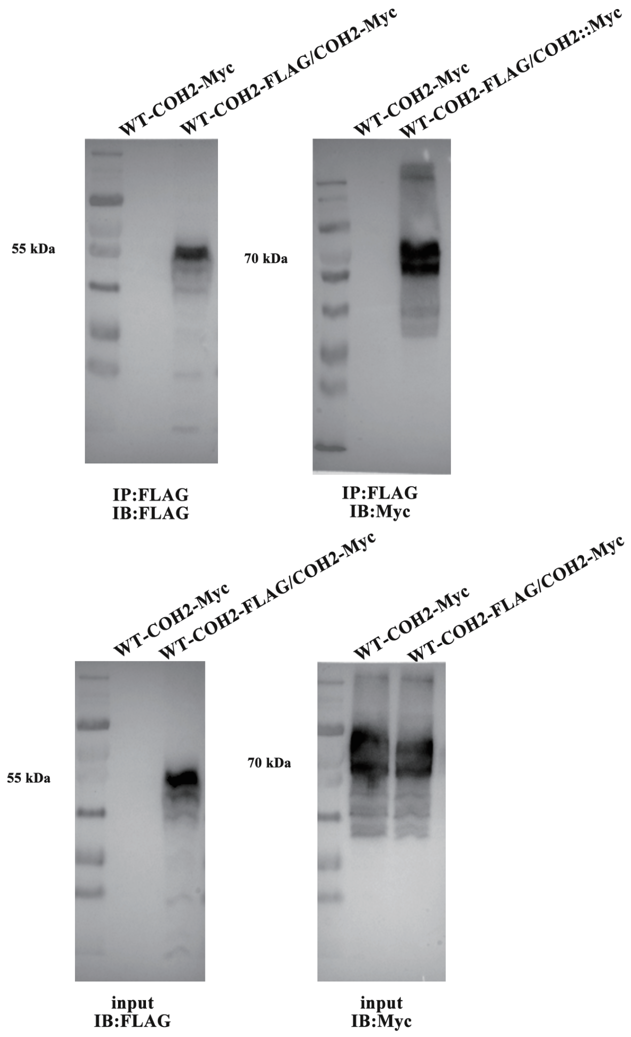

Fig. S6B

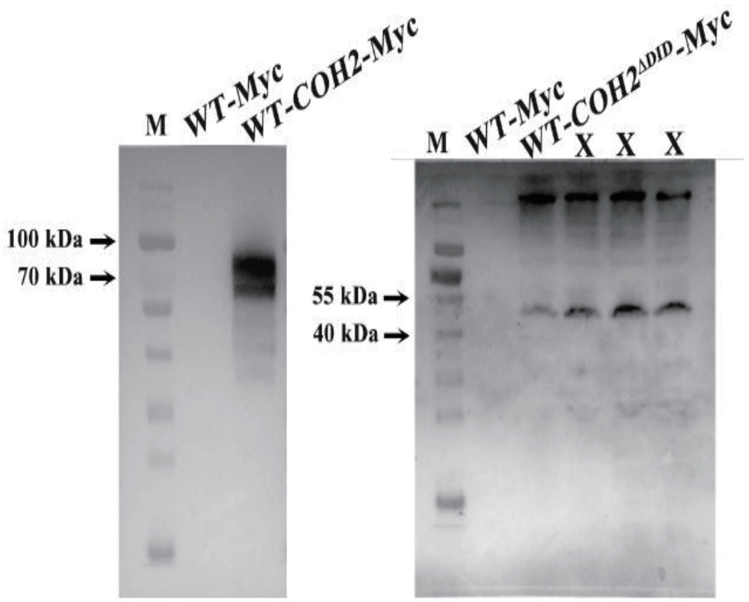

Fig. S6C

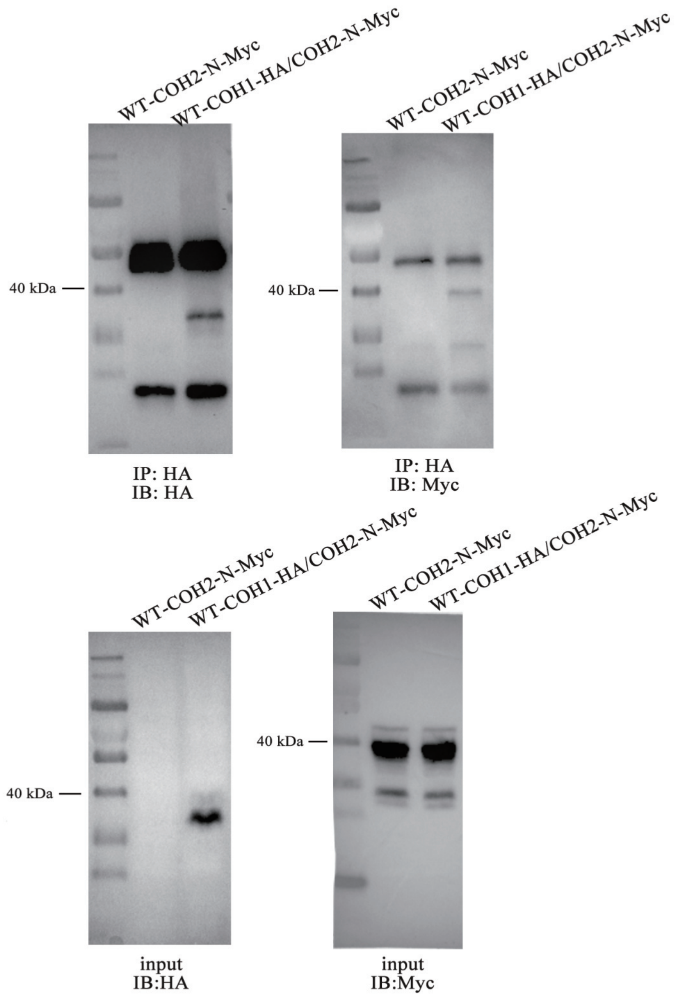

Fig. S6D

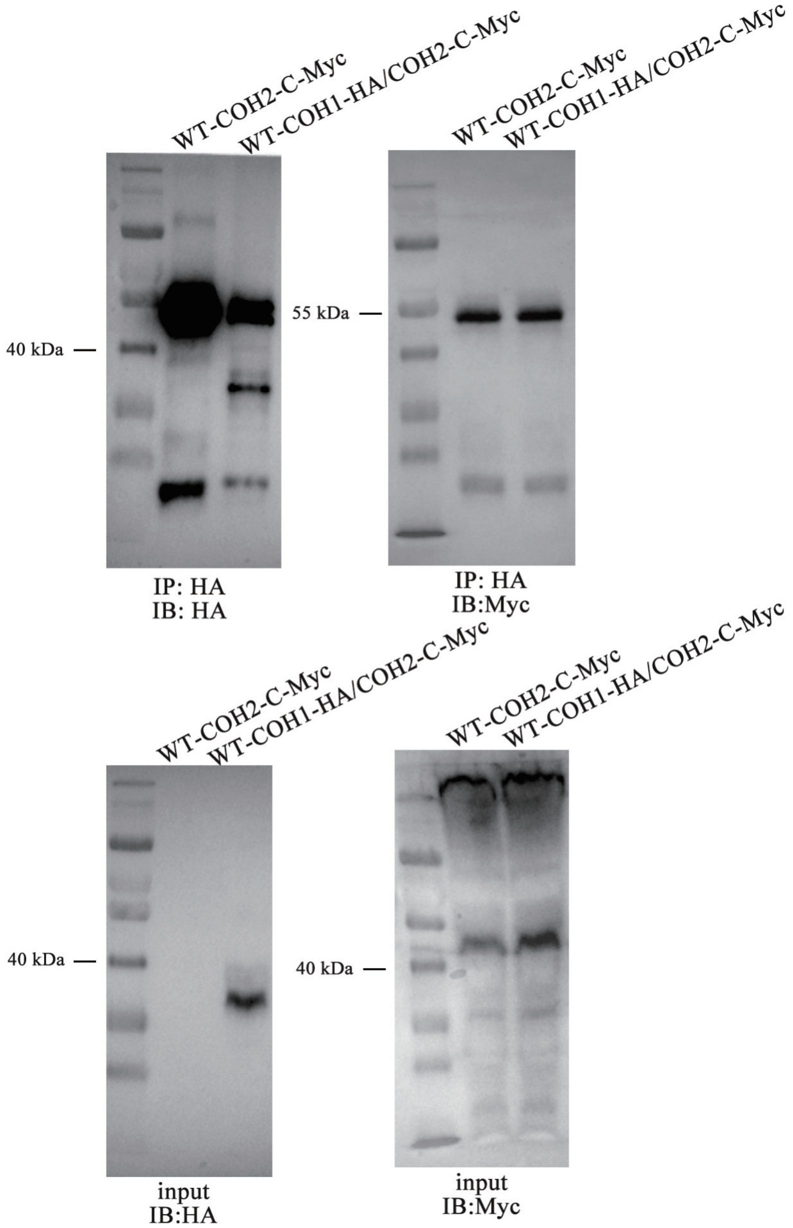

Fig. S8B

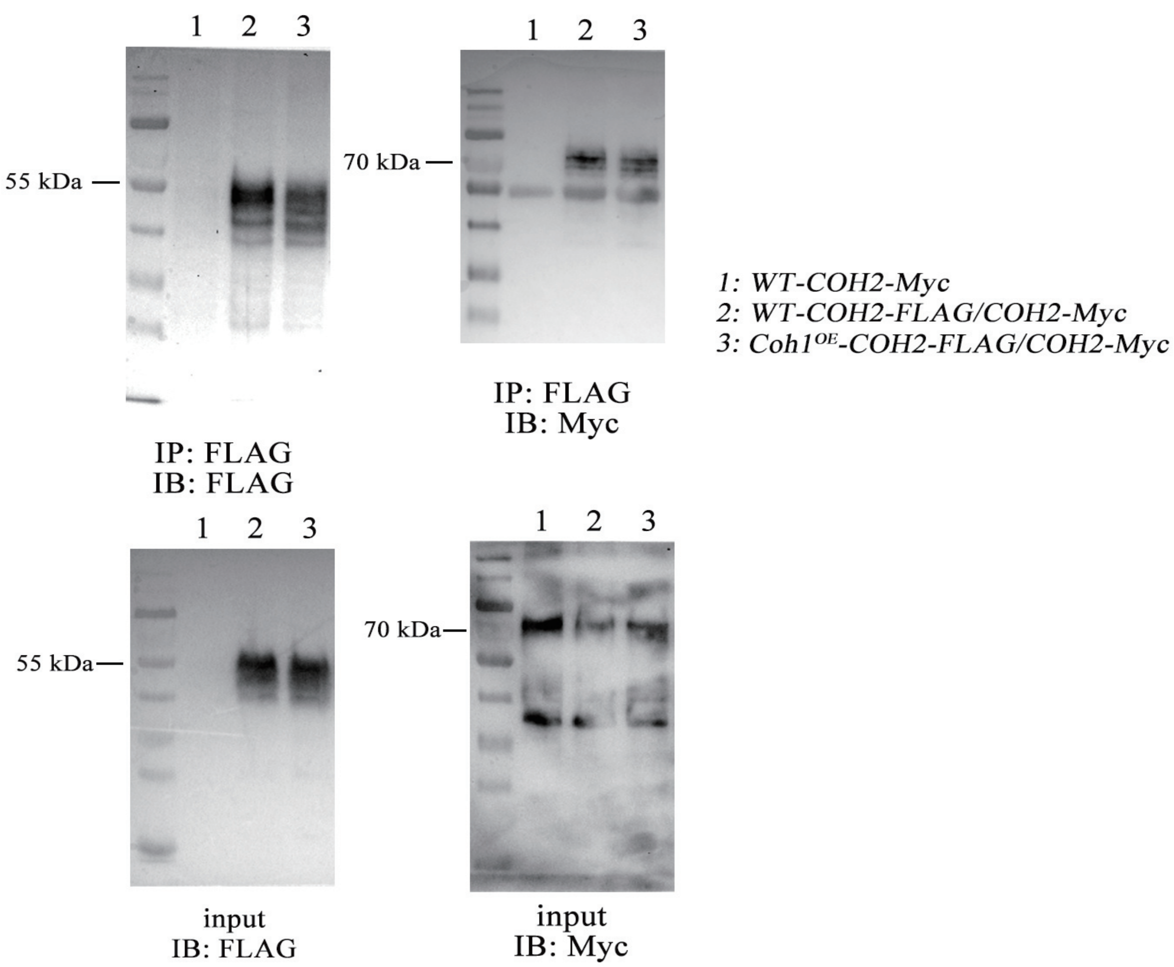

Fig. S8C

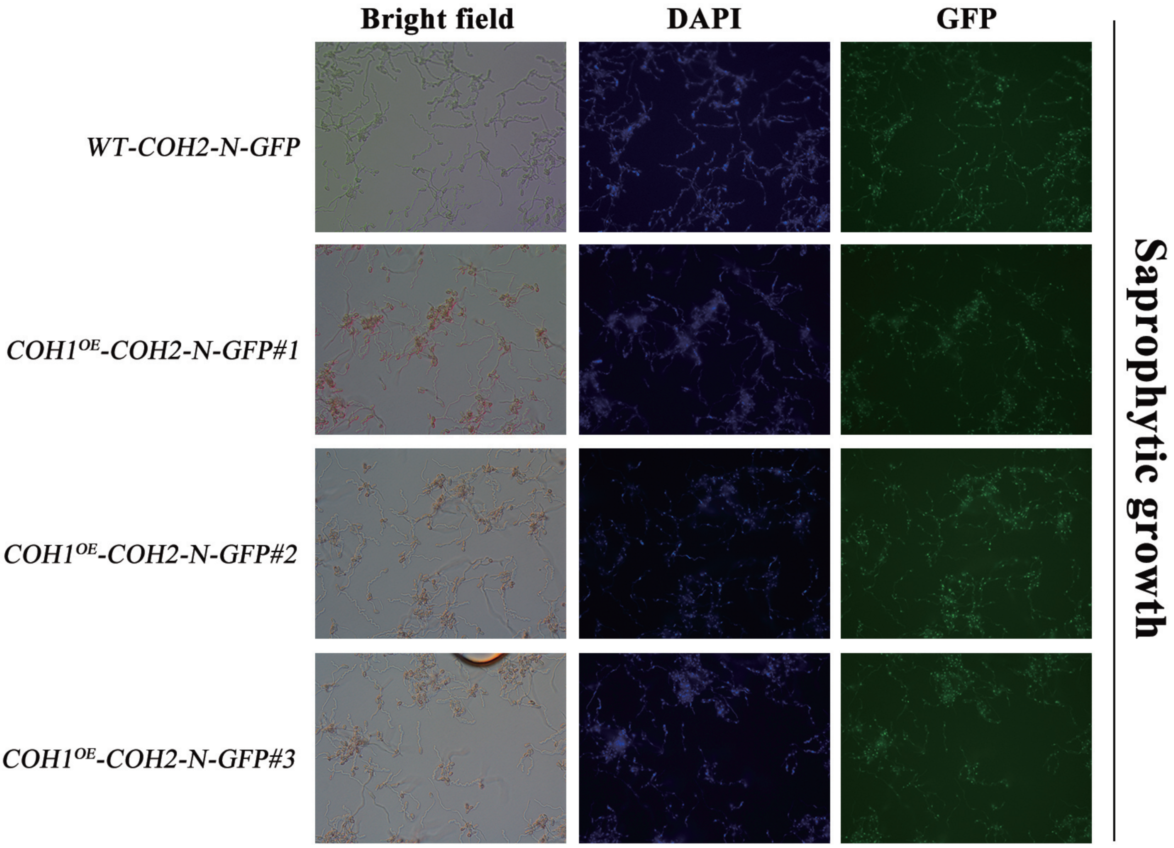

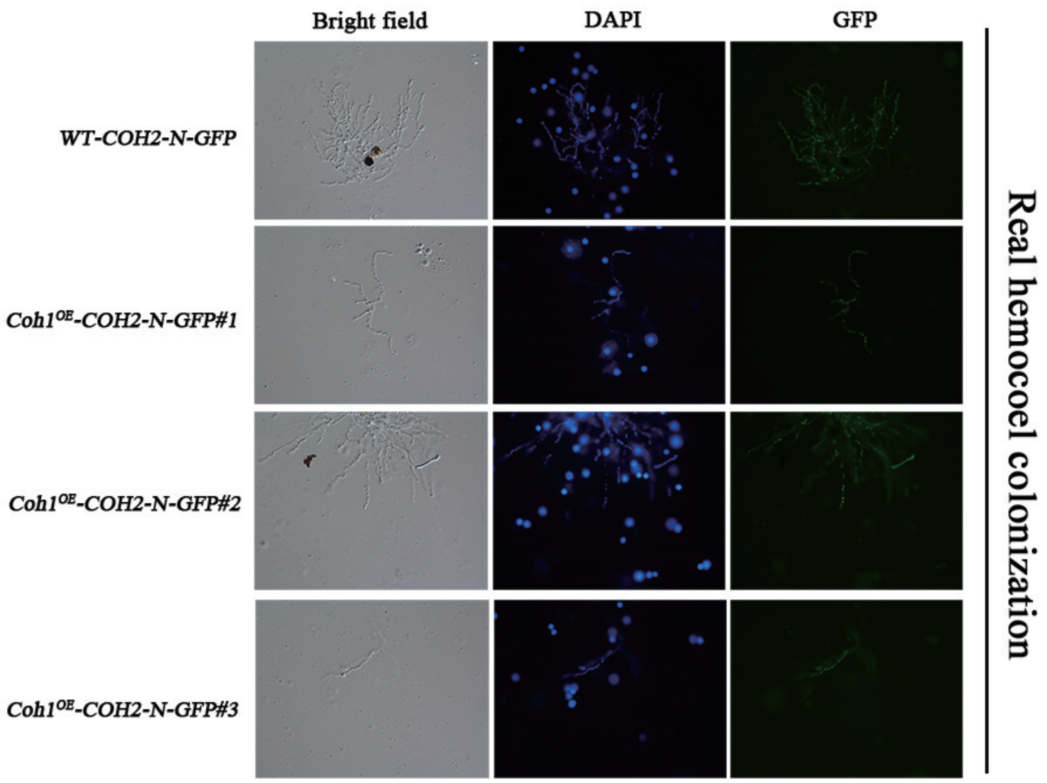
Bright field
DAPI
GFP
WT-COH2-N-GFP
Coh1^{OE}-COH2-N-GFP#1
Coh1^{OE}-COH2-N-GFP#2
Coh1^{OE}-COH2-N-GFP#3
Real hemocoel colonization

Fig. S8D

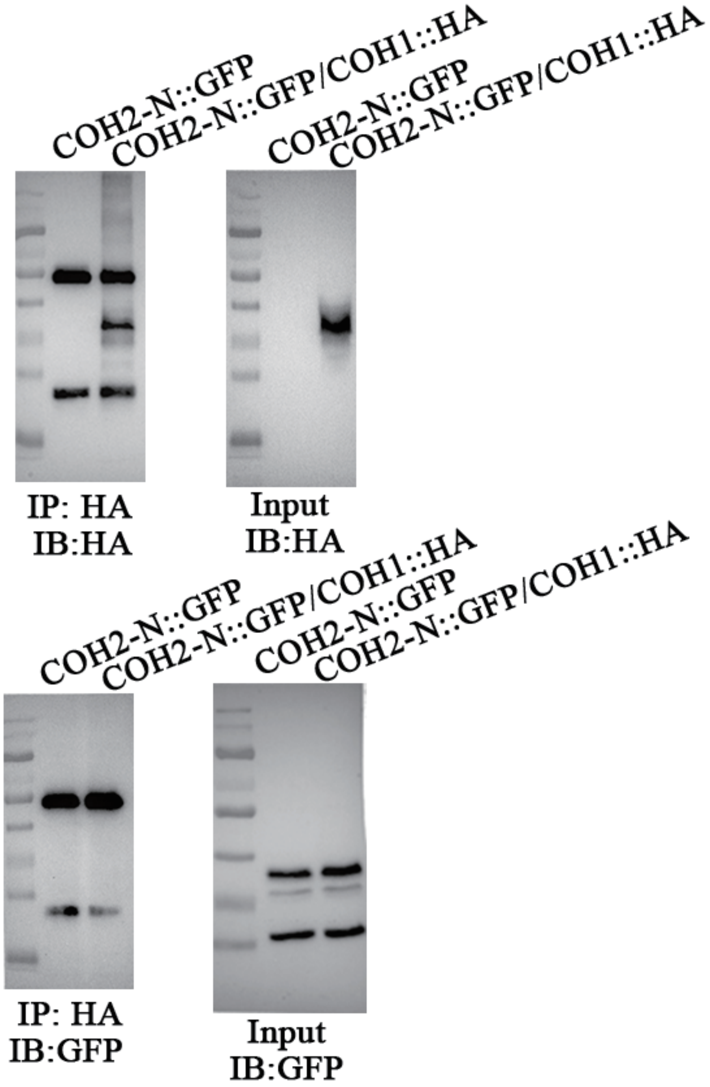
COH2-N::GFP
COH2-N::GFP/COH1::HA
COH2-N::GFP
COH2-N::GFP/COH1::HA
IP: HA
IB:HA
Input
IB:HA
COH2-N::GFP
COH2-N::GFP/COH1::HA
COH2-N::GFP
COH2-N::GFP/COH1::HA
IP: HA
IB:GFP
Input
IB:GFP

Fig. S9B

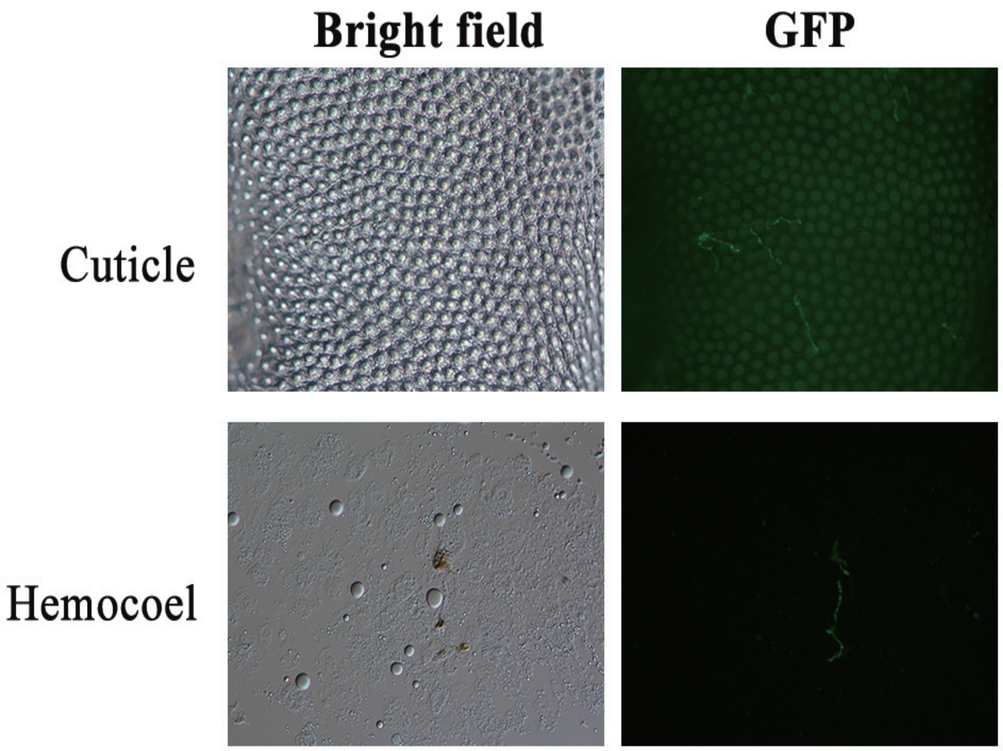

Fig. S11E

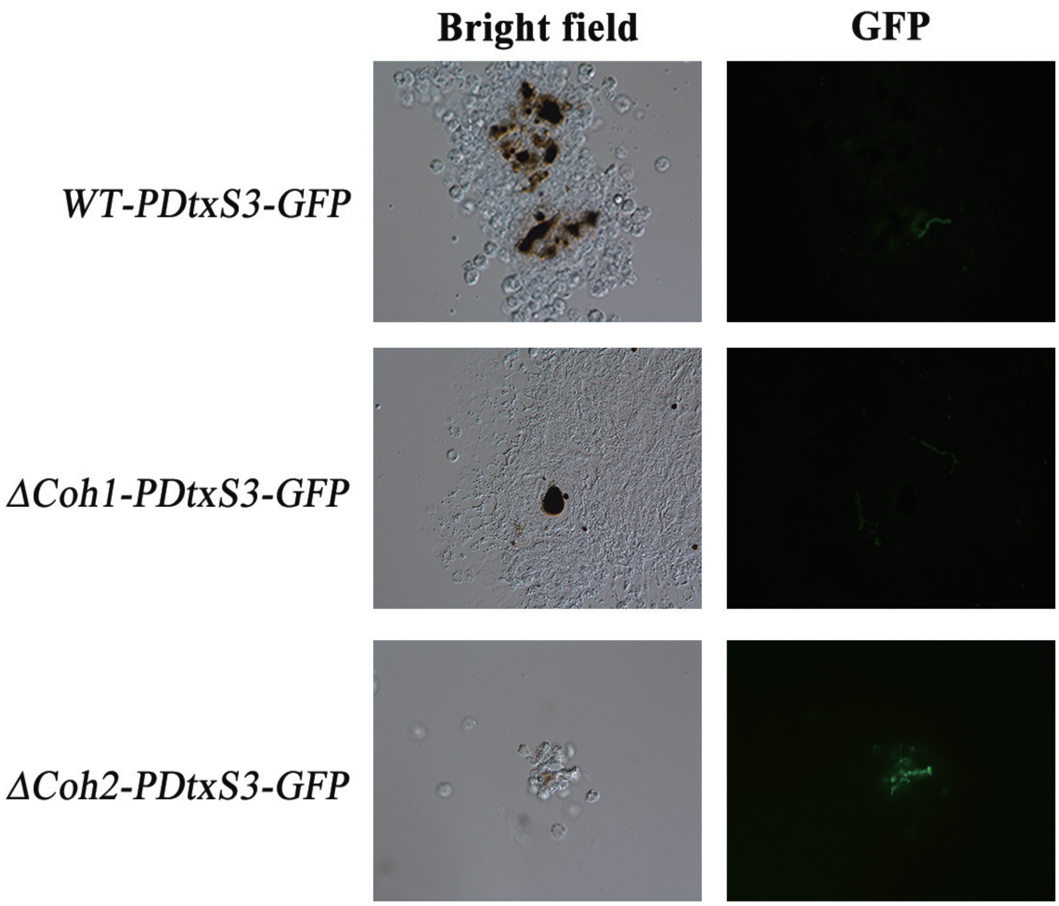

Supplement: S1 Raw Images — (PDF) [file pbio.3001360.s013.pdf]
