# Supplementary material for: A novel cascade allows Metarhizium robertsii to distinguish cuticle and hemocoel microenvironments during infection of insects
Source: PLoS Biol. 2021 Aug 4;19(8):e3001360. doi: 10.1371/journal.pbio.3001360 (PMC8366996; doi:10.1371/journal.pbio.3001360)
Supplement: S1 Table — (DOCX) [file pbio.3001360.s014.docx]

**Table S1. Primers used in this study.**

| **Primer** | | **Sequence** | | **Usage** |
| --- | --- | --- | --- | --- |
| Coh1-5-1 | GGGGACAGCTTTCTTGTACAAAGTGGAATCACGCCACTCACTAAG | | Deletion of *Coh1* | |
| Coh1-5-2 | GGGGACTGCTTTTTTGTACAAACTTGTACTGTTACAGGCAGAAC | |  |  |
| Coh1-3-1 | GGGGACAACTTTGTATAGAAAAGTTGTTCACTGATACCACGAAAC | |  |  |
| Coh1-3-2 | GGGGACAACTTTGTATAATAAAGTTGTCCCATCAAGTACTACTC | |  |  |
| Coh1-CF1 | TCGTTTGAGCATGTCAG | | Confirmation of the deletion of *Coh1* | |
| Coh1-CF2 | TTTCGTCCCATTCGATC | |  |  |
| Coh1-F | GGCCCGGGGCAATTCTCCTGGTAC | | Cloning the genomic clone of *Coh1* for complementation of *Coh1* deletion mutant | |
| Coh1-R | GGTCTAGACGTGTAATGCCGTCTTG | |  |  |
| Coh1-ORF-5 | GGGGATCCATGGCTTTCAACCTCTG | | Confirmation of the complementation of  *Coh1* deletion mutant and cloning the CDS of *Coh1* to construct the strain *Coh1^OE^* | |
| Coh1-ORF-3 | GGGATATCTTAGTACGATCGTCGAC | |  |  |
| Hdac1-5-1 | GGGAATTCTCACCGGCACCACTTTC | | Deletion of *Hdac1* | |
| Hdac1-5-2 | GGGAATTCCGAGCTACACACTTGAC | |  |  |
| Hdac1-3-1 | GGACTAGTGAGGATGCACGAGTTTG | |  |  |
| Hdac1-3-2 | GGGATATCACTTGGGTCTTCGTATG | |  |  |
| Hdac1-CF1 | AGGCATCGTGTGGTAAG | | Confirmation of the deletion of *Hdac1* | |
| Hdac1-CF2 | CAGGCATTCGAGCACTC | |  |  |
| Hdac1-F | GGACTAGTCCCCGCGAACTACATAG | | Cloning the genomic clone of *Hdac1* for complementation of *Hdac1* deletion mutant | |
| Hdac1-R | GGGATATCCATAAGTCGCAGCATTG | |  |  |
| Hdac1-ORF-5 | GGGGATCCATGACGATGAAACGTGG | | Confirmation of the complementation of  *Hdac1* deletion mutant and cloning the CDS of *Hdac1* to construct the strain *Hdac1^OE^* | |
| Hdac1-ORF-3 | GGGATATCTCAAGGGTTTTTTTTAACGCCGTTTGC | |  |  |
| Coh2-5-1 | GGGGACAGCTTTCTTGTACAAAGTGGAAATACGCGTACGCTCATG | | Deletion of *Coh2* | |
| Coh2-5-2 | GGGGACTGCTTTTTTGTACAAACTTGTGAGATGGTGAGATGATC | |  |  |
| Coh2-3-1 | GGGGACAACTTTGTATAGAAAAGTTGTTCATCAACAGCTCAGGAC | |  |  |
| Coh2-3-2 | GGGGACAACTTTGTATAATAAAGTTGTCCTGACGAGTCAATTTC | |  |  |
| Coh2-CF1 | CTCAGAGCTGGCACATG | | Confirmation of the deletion of *Coh2* | |
| Coh2-CF2 | GGTTGCTACGAAAATGG | |  |  |
| Coh2-F | GGTCTAGAAGGCGATTTGCACCATG | | Cloning the genomic clone of *Coh2* for complementation of *Coh2* deletion mutant | |
| Coh2-R | GGTCTAGACTTCATCTCGGGAGAAC | |  |  |
| Coh2-ORF-5 | ATGACGCGGTCATCATC | | Confirmation of the complementation of the *Coh2* deletion mutant | |
| Coh2-ORF-3 | TCATCGCGAGATGACGG | |  |  |
| pET28a-Coh1-F | GGGATATCATGGCTTTCAACCTCTG | | Cloning *Coh1*’s CDS for expression of the COH1 protein in *E. coli* | |
| pET28a-Coh1-R | GGGATATCTTAGTACGATCGTCGAC | |  |  |
| pET28a-Coh2-DBD-F | GGGAATTCATGACGCGGTCATCATC | | Cloning the DNA fragment encoding the N terminal of COH2 protein for expression in *E. coli* | |
| pET28a-Coh2-DBD-R | GGAAGCTTTTACGTCATGGACACGCCCA | |  |  |
| BD-Coh1-5 | GGCATATGATGGCTTTCAACCTCTG | | Cloning the CDS of *Coh1* for yeast two hybrid | |
| BD-Coh1-3 | GGGGATCCTTAGTACGATCGTCGAC | |  |  |
| AD-Coh2-5 | TGGCCATGGAGGCCAGTGAATTCATGACGCGGTCATCATC | | Cloning the CDS of *Coh2* for yeast two hybrid | |
| AD-Coh2-3 | GCCCACCCGGGTGGAATTCTCATCGCGAGATGACGG | |  |  |
| Coh1-HA-F | GGGGATCCATGGCTTTCAACCTCTG | | Cloning the CDS of *Coh1* to construct fusion protein COH1:: HA | |
| Coh1-HA-R | GGGGTACCGTACGATCGTCGACGTT | |  |  |
| Coh2-Myc-F | GGGGATCCATGACGCGGTCATCATC | | Cloning the CDS of *Coh2* to construct fusion protein COH2::Myc | |
| Coh2-Myc-R | GGGATATCTCGCGAGATGACGGCGT | |  |  |
| Coh2-FLAG-F | GGGGTACCATGACGCGGTCATCATC | | Cloning the CDS of *Coh2* to construct fusion protein COH2::FLAG | |
| Coh2-FLAG-R | GGGGTACCTCATCGCGAGATGACGG | |  |  |
| Coh2^ΔDID^-FLAG-F | GGGGATCCATGACGCGGTCATCATC | | Constructing the fusion protein COH2^ΔDID^::FLAG | |
| Coh2^ΔDID^-FLAG-R | GGGAATTCTCGCGAGATGACGGCGT | |  |  |
| Coh2-N-Myc-F | GGGGATCCATGACGCGGTCATCATC | | Cloning the CDS of N-terminus of *Coh2* to construct fusion protein COH2-N::Myc | |
| Coh2-N-Myc-R | GGGATATCCGTCATGGACACGCCCA | |  |  |
| Coh2-C-Myc-F | GGGGATCCATGTCGTCTCCTTACTC | | Cloning the CDS of C-terminus of *Coh2* to construct fusion protein COH2-C::Myc | |
| Coh2-C-Myc-R | GGGATATCTCATCGCGAGATGACGG | |  |  |
| Coh2-N-GFP-F | GGGGATCCATGACGCGGTCATCATC | | Cloning the CDS of N-terminus of *Coh2* to construct fusion protein COH2-N::GFP | |
| Coh2-N-GFP-R | GGGATATCCGTCATGGACACGCCCA | |  |  |
| RT-Coh1-F | GACTGGCCGATCAAGAGAAG | | qRT-PCR for *Coh1* | |
| RT-Coh1-R | GCTCAAAAGTAGAGGCGGTG | |  |  |
| RT-Hat1-F | AAGTGGCAGTCGAAAGGCTA | | qRT-PCR for *Hat1* | |
| RT-Hat1-R | TAGCAAGAGTGGGCTCTGGT | |  |  |
| RT-Hdac1-F | TGCCCCAGATCTACATTTCC | | qRT-PCR for *Hdac1* | |
| RT-Hdac1-R | GGGAGTCACGTTGATGACCT | |  |  |
| RT-Coh2-F | GCGACTGGAAAGAGAACTGG | | qRT-PCR for *Coh2* | |
| RT-Coh2-R | CTGTTGGGGTCGAGTAAGGA | |  |  |
| DtxS1-RT-F | CGAGGTCGAGTTCAGAGTCC | | qRT-PCR for *DtxS1* | |
| DtxS1-RT-R | AGCTGCTCCATCCTGTCTGT | |  |  |
| DtxS2-RT-F | GACCAACCCTTATTGCAGGA | | qRT-PCR for *DtxS2* | |
| DtxS2-RT-R | CATGATAGGCCAGGTTTCGT | |  |  |
| DtxS3-RT-F | CCGTTGGCATCTGGTAGACT | | qRT-PCR for *DtxS3* | |
| DtxS3-RT-R | GGCTGTTACCCGTCGATTTA | |  |  |
| DtxS4-RT-F | TCCTTGGGATTCTCAACACC | | qRT-PCR for *DtxS4* | |
| DtxS4-RT-R | GACAACGAGGGATGTCAGGT | |  |  |
| 10457-RT-F | CCAGATGCTTGACCAGATGC | | qRT-PCR for *MAA_10457* (Siderophore-iron transporter) | |
| 10457-RT-R | TTGCCGAGGTAGTAGTTGGG | |  |  |
| 00990-RT-F | AGAGACTGCCATAGGAGTGC | | qRT-PCR for *MAA_00990* (Lcc4) | |
| 00990-RT-R | TAGCCAAACTCCCGGATTGT | |  |  |
| 00272-RT-F | GAGCGTCACTATCACCAGCA | | qRT-PCR for *MAA_00272* (histone methyltransferase) | |
| 00272-RT-R | CGATCCGGTGTACGAAAGAT | |  |  |
| 2065-RT-F | TTCATGAGGTCCTTCGTTCC | | qRT-PCR for *MAA_02065* (histone deacetylase) | |
| 2065-RT-R | TCATCGTGGCTAGCTTTGTG | |  |  |
| 4246-RT-F | ATTCAGCTGCACGGAAGTCT | | qRT-PCR for *MAA_04246* (histone deacetylase) | |
| 4246-RT-R | GTCTTAGGCGTCCAATTCCA | |  |  |
| 4679-RT-F | ACCGCAACATCGATAAGGTC | | qRT-PCR for *MAA_04679* (histone acetyltransferase) | |
| 4679-RT-R | TAGCAGCGGCTGGTTATCTT | |  |  |
| 4734-RT-F | GATGCTGGGTTCAAGGTTGT | | qRT-PCR for *MAA_04734* (histone acetyltransferase) | |
| 4734-RT-R | GCCGTCTGTTCTAAAGGCTG | |  |  |
| 4846-RT-F | ACCTCAGAGGCCAACATCAC | | qRT-PCR for *MAA_04846* (histone deacetylase) | |
| 4846-RT-R | CCCGAGCAAAAGAGACTGAC | |  |  |
| 5326-RT-F | GACGACACTGCCTCTGATGA | | qRT-PCR for *MAA_05326* (histone deacetylase) | |
| 5326-RT-R | AGCAGCAACCAGTAGGGAGA | |  |  |
| 5985-RT-F | GGCATCTCTACGCGATAAGC | | qRT-PCR for *MAA_05985* (histone deacetylase) | |
| 5985-RT-R | GGCCTTCAATCTTTGGATGA | |  |  |
| 9293-RT-F | TCTACGAGTGTGATGCTGGC | | qRT-PCR for *MAA_09293* (histone methyltransferase) | |
| 9293-RT-R | GTTGCTTCTAACGCCGTAGC | |  |  |
| 10199-RT-F | ACGGGGTCATCTGCGATATT | | qRT-PCR for *MAA_10199* | |
| 10199-RT-R | GCGTTGAATTGCAGAAAGGC | |  |  |
| 10350-RT-F | TTCTTCCTATTCACGCACGC | | qRT-PCR for *MAA_10350* | |
| 10350-RT-R | GATGCTCCATGACACGACAC | |  |  |
| 10456-RT-F | GTGGTCAAGTTGGTGCAGAG | | qRT-PCR for *MAA_10456* | |
| 10456-RT-R | CGGCCTTAGGTAGTGGTCTT | |  |  |
| ChIP-Coh1-F | AGGCCACCACAATATCGACT | | ChIP-qPCR analysis of *Coh1* | |
| ChIP-Coh1-R | GAATACACGGACGGCCAAAA | |  |  |
| ChIP-Hdac1-F | TCACTCACTCGCTCTGTTGG | | ChIP-qPCR analysis of *Hdac1* | |
| ChIP-Hdac1-R | GTTGCACGCTCTTCATACCA | |  |  |
| ChIP-DtxS1-F | TGGGGAGAACTTGAAGGATG | | ChIP-qPCR analysis of *DtxS1* | |
| ChIP-DtxS1-R | CGGTTTTTGGTCCATTTTTG | |  |  |
| ChIP-DtxS2-F | GGGTTGAGTGAAAGGGTCAA | | ChIP-qPCR analysis of *DtxS2* | |
| ChIP-DtxS2-R | GCAAAAGGCCTTGTTTTCAC | |  | |
| ChIP-DtxS3-F | GCTAAACGGCACTTTGGTCA | | ChIP-qPCR analysis of *DtxS3* | |
| ChIP-DtxS3-R | GTTGATTCGTGGTGGGTGTC | |  |  |
| ChIP-DtxS4-F | AGTGCAAGGGGGTGTAAGTG | | ChIP-qPCR analysis of *DtxS4* | |
| ChIP-DtxS4-R | CGCTGCTGATTAGGTTTTCC | |  |  |
| ChIP-4430-F | AGAACCATGCCCGAGTAACA | | ChIP-qPCR analysis of *MAA_04430* | |
| ChIP-4430-R | TATGCCACCACGTTACCCAT | |  |  |
| ChIP-10199-F | AGGGAAGCTGGTCAACATGA | | ChIP-qPCR analysis of *MAA_10199* | |
| ChIP-10199-R | CAGGAATGCTTGTCGTAGGC | |  |  |
| ChIP-10350-F | ATGTCGTAGCCGCAAATCAC | | ChIP-qPCR analysis of *MAA_10350* | |
| ChIP-10350-R | ATTGTCAAGTACCAAGCCGC | |  |  |
| ChIP-10456-F | GACCCTCCTCAAGACCAGAC | | ChIP-qPCR analysis of *MAA_10456* | |
| ChIP-10456-R | GCAGCAACTCTTCTTCGTGT | |  |  |
| Southern-bar-F | CCATCGTCAACCACTAC | | Cloning the sequence of the herbicide resistance gene *Bar* as a DNA probe for Southern blotting. | |
| Southern-bar-R | CAGAAACCCACGTCATG | |  |  |
| ITS-F | TACGCCCCTCAAGTCCCCTG | | qRT-PCR for fungal burden analysis | |
| ITS-R | GGCTCCTGTTGCGAGTG | |  | |
| P-Hat1-F | ACACAGGAAACAGCTATGACATGATTACGAATTCCGTGAAATGATTTCCTG | | Constructing a strain expressing GFP driven by *Hat1* promoter | |
| P-Hat1-R | GCCCTTGCTCACCATGATATCGAATTCCATGACCCTTGCTTCAT | |  |  |
| P-Hdac1-F | ACACAGGAAACAGCTATGACATGATTACGAATTCATGGAGTACTCCGTATG | | Constructing a strain expressing GFP driven by *Hdac1* promoter | |
| P-Hdac1-R | GCCCTTGCTCACCATGATATCGAATTCGAAGGAAATAGTTGTGT | |  |  |
| P-Coh2-F | ACACAGGAAACAGCTATGACATGATTACGAATTCGTCGCCATTCTACACAC | | Constructing a strain expressing GFP driven by *Coh2* promoter | |
| P-Coh2-R | GCCCTTGCTCACCATGATATCGAATTCGGTTGTGAAGGCTGGAT | |  |  |
| P-10199-F  P-10199-R  P-DtxS3-F  P-DtxS3-R  PCoh2-FLAG-F  PCoh2-FLAG-R  Gal-RT-F  Gal-RT-R  Defensin-RT-F  Defensin-RT-R  18S-RT-F  18S-RT-R | CCGTCCGTCTCTCCGCATGCACTAGTGTAGTAGCGGCCATCTG CCGTCCGTCTCTCCGCATGCACTAGTGTAGTAGCGGCCATCTG  ACACAGGAAACAGCTATGACATGATTACGAATTCGCTCGTTGCTGTATATG  GCCCTTGCTCACCATGATATCGAATTCGGTTACCAGAACTTCAA  TGCATGCTCTCACACTAGTGACTGATATCGTCGCCATTCTACACAC  ATCGACCGACGGAATTGAGGATATCGCATTGCAGATGAGCTG  CTCGTAAAATACACATCCGGGG  CTCGTAAAATACACATCCGGGG  CGTCACCACAAAATGCTGTACA  CTACTCCTCGCACCAACAA  CGCGCTACACTGAAGGAATC  AAGGGCAGGGACGTAATCAA | | Constructing a strain expressing GFP driven by *MAA_10199* promoter  Constructing a strain expressing GFP driven by *DtxS3* promote  Constructing the strain*ΔCoh2-PCoh2-COH2-FLAG*  qRT-PCR for gallerimycin  qRT-PCR for defensin  qRT-PCR for 18S rRNA | |
