# Supplementary material for: A novel cascade allows Metarhizium robertsii to distinguish cuticle and hemocoel microenvironments during infection of insects
Source: PLoS Biol. 2021 Aug 4;19(8):e3001360. doi: 10.1371/journal.pbio.3001360 (PMC8366996; doi:10.1371/journal.pbio.3001360)
Supplement: Attachment — Submitted filename: Response to Reviewers.docx [file pbio.3001360.s015.docx]

Dear editors,

Thank you and reviewers very much for valuable comments on our paper. We have amended the manuscript as suggested. Our point-to-point responses to reviewer and editor comments are pasted below. Several changes are highlighted here for your consideration.

1. In response to the comment about “monitoring a differential response to two different insect species”, in the revised manuscript, we studied the responses of *M. robertsii* to two different microenvironments (the cuticle and the hemocoel) within the same insect species (*Galleria mellonella* larvae).
2. According to the suggestions from reviewer #3, we validated many results in real infection conditions. For example, we used Histoimmunochemical staining to assay the impact of the expression of COH1 on the level of the COH2 protein in fungi collected from the real hemocoel of the insects infected by *M. robertsii*.
3. According to the academic editor’s suggestions, we revised our figures for better presentation and set appropriate controls for Co-IP experiments.

Best regards

Sincerely yours

Weiguo Fang

**Point-to-point response to reviewer and editor comments**

**Editor comments:**

**Comment 1:** The reviews of your manuscript are appended below. You will see that the reviewers find your work potentially interesting, however they also raise several concerns that would need to be address if we decide to pursue the manuscript for publication. Both Reviewers 1 and 2 have relatively minor points, but Reviewer 3 is more critical and thinks you cannot formally exclude that you are monitoring a differential response to two different insect species, locust in the case of cuticle and moth for the hemolymph. After consulting with the academic editor, we deem this point essential for publication and you will need to provide a convincing response to address this issue. Ideally if you want to study differences to different tissues it would have to be in the same host.

**Response:** According to this suggestion, we studied the responses of *M. robertsii* to two different microenvironments (the cuticle and the hemocoel) within the same insect species (*Galleria mellonella* larvae). In the first submission locust cuticle and *G. mellonella* hemolymph were used; in the revised manuscript, both the cuticle and the hemolymph were from the *G. mellonella* larvae.

**Comment 2:** In addition, this reviewer thinks you should validate the model in real infection conditions and use antibodies against COH2 to show that the expression of Coh1 leads to the degradation of COH2 during hemolymph colonisation.

**Response:** The *M. robertsii* strain ARSEF2575 used in this study kills insects by fungal growth and toxins; the biomass (hyphal bodies) in the real hemocoel of the infected insects (the real infection conditions) is extremely low, and it is impossible to retrieve enough fungal biomass to preparing sufficient proteins for Western blotting analysis. So, we used the hemocyte-containing hemolymph as an approximation to the hemocoel in the real infection conditions. However, we think this comment is very valuable, we thus used Histoimmunochemical staining to assay the impact of the expression of COH1 on the level of the COH2 protein in the mycelium collected from the real hemocoel of the insects infected by *M. robertsii*. New data are included in the revised manuscript (Fig. 3D).

**Comment 3:** This reviewer also suggests other experiments to demonstrate that the Coh1 phenotype is not due to a secondary mutation, which would be essential to address and requires either an independent mutant or genetic complementation, and the generation of a Coh1-Coh2 double mutant, which we don’t consider a requirement for publication.

**Response:** Although the editors think the experiments suggested by the reviewer 3 in this comment are not required to be done for publication, we still tried our best to address this comment in the revised manuscript. For the secondary mutation issue, we constructed the *Coh1*-overexpressing strain *Coh1^OE^* according to the reviewer’s suggestion; we found that *ΔCoh1* phenotype is not due to a secondary mutation (see below for our detailed response to the reviewer’s comment). We also generated the Coh1-Coh2 double deletion mutant, and the data about this strain were included in the revised manuscript.

**Comment 4:** The academic editor has also mentioned that the quality of the figures should be improved, and notes an excessive use of histograms over scatterplots and poorly controlled experiments such as the coIP in Figure 3B.

**Response:** In this manuscript, there is a large amount of data from qRT-PCR and ChIP-qPCR analyses, and actually such type of data is usually presented with histograms in many other research articles. To address this comment, in the revised manuscript we used scatterplots to present LT_50_, fungal burden, enzyme activity and amounts of amino acids and peptides (Fig 1B; Fig S2D, F, G; Fig S3D, E; Fig. S9G, H; Fig S11H, I, J, K).

According to the academic editor’s comment, we set appropriate negative controls in the Co-IP analysis (Fig 3B; Fig S6A, C, D; Fig S8B).

**Reviewers’ comments
Rev. 1:**
Cuticular penetration and hemocoel colonization are cellular processes and events critical for the pathogenesis of a fungal insect pathogen. This interesting manuscript presents massive data from technically robust experiment performed to characterize a novel cascade that regulates the pathogenesis of Metarhizium robertsii. The cascade comprises a pair of core proteins, namely the regulatory protein COH1 expressed exclusively in hemocoel colonization and the transcription factor COH2 localized in nucleus. The COH1-coding gene was evidently required for the fungal virulence via normal cuticle infection and transcriptionally mediated by HADC1 and HAT1, two key enzymes regulating histone H3 acetylation in the promoter region of the gene. COH1 was found interacting with the N-terminus of COH2 but not affecting an entry of COH2 into the nucleus. The interaction can reduce COH2 stability through a proteasome-dependent pathway and derepress the COH2-mediated repression of genes involved in hemocoel colonization. COH2 activates the expression of cuticle degradation-related genes during normal cuticle infection by directly binding to their promoter regions but those genes are repressed by COH1 during hemocoel colonization. These findings unveil a novel mechanism underlying the fungal adaptation to the microenvironments insect cuticle and hemocoel encountered during the normal infection and after cuticular penetration respectively and offer an in-depth insight into the impressive roles of COH1 and COH2 in coordinating the processes of cuticle infection and intrahemocoel propagation post-infection.

**Comment 5:** Overall, all data are well organized and presented. The manuscript needs a careful revision to improve clarity and conciseness.

**Response:** according to this comment and comments from other two reviewers, we thoroughly revised the manuscript for better clarity and conciseness.

**Comment 6:** Labels and legends in most figures are too busy or not proportional to chart size. Many bar charts can be improved by proper adjustments of bar-to-bar spaces, labels and legends. Using log10 scale in some bar charts may help to show big differences of gene transcripts between strains.

**Response:** According to this comment, we revised the figures (Fig1, 3, 4, 5 and Fig S1, S2, S3, S7, S8, S10) for better presentation.

**Rev. 2:**
The manuscript entitled "Unveiling a novel cascade regulating the response of Metarhizium robertsii to distinct microenvironments during infection of insects" reports the functional characterization of two interacting transcription factors (COH1 and COH2) regulating gene expression of a parasitic fungus on the cuticle or within the hemocoel of the infected host insect. COH1 was expressed upon epigenetic repression conferred by the histone deacetylase and the histone acetyltransferase. The study is interesting and the authors have used a considerable spectrum of methods to elaborate a convincing data set. I appreciate the extended spectrum of experimental approaches such as yeast-two-hybrid system to determine interactions between COH1 and COH2. I have some minor comments to improve the manuscript constructively:

**Comment 7:** In the introduction, the authors should mention the important role of proteolytic enzymes during penetration of the cuticle. Some of these enzymes including metalloproteases have been recognized to operate as virulence factors whose control is important.

**Response:** Information added, and a new literature cited.

**Comment 8:** The authors should discuss what factors could trigger the expression of COH1 in the hemocoel. The entity of recent work implicates that transcriptional reprogramming in *M. robertsii* and other entomopathogenic fungi during infection is more plastic than previously thought and that they respond in a specific manner to host defense molecules.

**Response:** The COH1 expression in the hemocoel is regulated by HDAC1, which is in turn controlled by HAT1. In the revised manuscript, we found that the reduction in Hat1 expression in the hemocoel was not due to the feedback regulation by COH1 and COH2. During the microenvironmental transition from the cuticle to the hemocoe*l, M. robertsii* encounters vast environmental changes with the host defense, nutrients, osmolarity and availability of oxygen. The triggers for the transcriptional reprogramming controlled by the regulatory cascade could be multifactorial and remain to be resolved. This is discussed in the revised manuscript.

**Rev. 3:
Comment 9:** In this study, Weiguo Fang and coworkers focus on a gene, Coh1, found to be expressed in conditions thought to reproduce the ones happening during the colonization of insect hemocoel by the entomopathogenic fungus Metarhizium robertsii, that is, growth in collected cell-free Galleria mellonella hemolymph. They check by RTqPCR that it is not expressed when the fungus is grown in SDY medium nor in medium containing locust cuticle as the sole carbon and nitrogen source. They link this specific pattern of expression to a modification of epigenetic marks that would result from the decreased expressions of the Hat1 histone acetyl transferase and Hdac1 histone deacetylase genes at this specific stage. The involvement of enzymes with two opposing activities would reflect the positive action of HAT1 on Hdac1 transcription, thereby resulting in the expression of Coh1 only when the conidia are incubated with hemolymph. Coh1 deletion mutants display a strongly decreased virulence when topically applied to G. mellonella larvae. The direct injection of conidia led to a much milder phenotype of decreased virulence. Of note, the three complementation constructs expressing the wild-type Co h1 gene from distinct genomic locations (random integration?) failed to rescue the mutant phenotype, possibly because of the different chromatin environment in which the wild-type gene is inserted in each transformant. The authors next use a strategy that makes little common sense to identify a bZIP transcription factor, COH2, that happens to interact with COH1.They show that the binding of COH1 to COH2 destabilizes the transcription factor that likely gets degraded through the proteasome. The authors next show that COH2 is involved in pathogenicity, the corresponding gene being required for piercing through the insect cuticle. It is thus difficult to establish whether this gene also plays a role in the phase of hemocoel invasion, as it can only be addressed by injecting conidia; they find a decreased virulence phenotype that is as weak as that displayed by Coh1 mutants, even though it was not expected to as COH2 is supposedly unstable during this stage of the infection.

They characterize in detail the DNA binding motif of COH2 and use this knowledge to interpret RNAseq experiments performed during "hemocoel colonization" by the two mutants vs. wild-type. They conclude that Coh1 is required to repress the expression of some Coh2 regulated genes involved in piercing the cuticle through its enzymatic degradation as well as to stimulate the expression of genes encoding toxins that are presumably required for successful invasion of the host.

While this study is rather strong on the molecular biology of the Coh1 and Coh2 genes, it sorely lacks a validation of the proposed model under real infection conditions. The authors over-rely on the postulate that the artificial conditions they use accurately reproduce the actual situation occurring in vivo during infection. This is already reflected in the title and throughout the manuscript when for instance employing the term "hemocoel colonization", a clear overstatement. For instance, the authors cannot formally exclude that what they are monitoring is a differential response to two different insect species, locust in the case of cuticle and moth for the hemolymph. More importantly, the hemolymph is collected from naive larvae and lacks immune cells: it cannot be excluded that the expression of genes if the fungus is altered by the exposure host defenses. While this reviewer understands the practical issues that lead to use this set-up, it is absolutely indispensable to validate the findings in vivo at different stages of the infection. They thus have to monitor the steady-state levels of Coh1, Coh2, Hat1, Hdac1, and representative genes regulated by Coh1 or Coh2 under in vivo conditions. Of note, they likely could retrieve hyphal bodies from the hemolymph of infected larvae. The current title is also inaccurate as long as these points have not been addressed.

**Response:** We agree with the reviewer on this comment that findings obtained from *in vitro* system should be validated *in vivo*. It is true that it is better to use the same insect species to study the difference in *M. robertsii* responses to two different microenvironments. To address this comment, we did two experiments.

1. In the first submission, the locust hindwings and the hemolymph from *G. mellonella* larvae were used. In the revised manuscript, the cuticle and the hemolymph were from the same insect species (the *G. mellonella* larvae). We would like to point out that the larval hemolymph we prepared in this study contained hemocyte. The freshly hemolymph was collected and immediately mixed with an anticoagulant solution to prevent cell coagulation, so the hemocytes remained in the prepared hemolymph. The details about how to prepare the hemolymph and the cuticle of the *G. mellonella* larvae were provided in the revised manuscript. The data obtained with the *G. mellonella* cuticle was not different from that with the locust hindwings, and all data about the *G. mellonella* cuticle were included in the revised manuscript.

For RNA-seq analysis of genes regulated by *Coh2* during the cuticle penetration, we still used the data with the locust hindwing because it is easy to get a large amount of locust hindwings from commercially available locusts, on which *M. robertsii* can grow to obtain enough fungal biomass for RNA-seq analysis. However, we validated the RNA-seq data on the *G. mellonella* cuticle by analyzing several representative genes using qRT-PCR. The new data were included in the revised manuscript.

2. According pointed out by the reviewer, there were practical issues that lead to use approximations to the real infection conditions. However, we followed the reviewer’s suggestions and validated the findings obtained with the approximation to the hemocoel colonization in the real hemocoel of the infected insects. *M. robertsii* kills insects by fungal growth and toxins, and the fungal biomass in the real hemocoel of the infected insects is extremely low, so it is impossible to retrieve enough hyphal bodies for preparing sufficient fungal proteins for Western blotting, RNA for qRT-PCR, and DNA for ChIP-qPCR. We thus used two strategies to validate the results obtained from the *in vitro* experiments in the real hemocoel of the infected insects.

First, the expression of a gene during cuticle penetration and in the real hemocoel of the infected insects was analyzed by tracing the GFP fluorescent intensity in the cells of the strain with the *gfp* gene driven by the promoter of the studied gene. Using this strategy, we studied *Coh2*, *Hat1*, *Hdac1*, and several COH1/COH2 regulated genes including protease and destruxin biosynthesis genes. The new results obtained from the *in vivo* experiments were consistent with those from the i*n vitro* experiments, and the data were shown in Fig. 1E, Fig. 5G, Fig. S9B, and S11E in the revised manuscript. Since the native chromosomal position is important for the activity of *Coh1* promoter, the GFP strategy is not suitable for the *in vivo* analysis of *Coh1* expression. So, we used regular RT-PCR to investigate whether *Coh1* was expressed in the real hemocoel of infected insects using the total RNA prepared from the insects infected by the WT strain, which contained RNAs from *M. robertsii* in the hemocoel and the insects. These data were included in the Fig. 1A in the revised manuscript.

Second, we used histoimmunochemical staining to assay the impact of the expression of COH1 on the level of the COH2 protein in the mycelium collected from the real hemocoel of the infected insects. The data showed that the expression of COH1 did reduce the level of COH2 protein in the real hemocoel, which is shown in the Fig. 3D.

**Comment 10:** 2. The general model is that the expression of Coh1 leads to the degradation of COH2 during "hemolymph colonization". It would be ideal to demonstrate this in vivo using an antibody raised against COH2. At the very, least, the authors could check the tagged COH2 construct comparing wild-type vs. Coh1 deletion mutants after infection.

**Response:** As stated above, it is impossible to retrieve enough fungal biomass from the real hemocoel of the infected insects to prepare sufficient protein for Western blot analysis, so we used histoimmunochemical staining to assay the impact of the expression of COH1 on the level of the fusion protein COH2׃׃FLAG in the fungal propagules collected from the real hemocoel. The COH2::FLAG protein was found to be more abundant in the strain *ΔCoh1-COH2–FLAG* than the *Coh1* expressing strain *WT-COH2-FLAG*, which in turn had more COH2::FLAG protein than the *Coh1*-overexpressing strain *Coh1^OE^-COH2–FLAG.* Therefore, COH1 did reduce the level of COH2 protein in the real hemocoel of the infected insects, and the new data were shown in the Fig. 3D in the revised manuscript.

**Comment 11:** What happens to cuticle penetration and virulence in the topical infection model when Coh1 is overexpressed? Conversely, what happens when Coh2 overexpressing conidia (in wild-type or Coh1 mutant background) are injected into G. mellonella larvae?

**Response:** We constructed the *Coh1*-overexpressing strain *Coh1^OE^*. It is assumed that overexpression of Coh1 could reduce the ability of the WT strain to penetrate cuticle because COH1 repressed the expression of cuticle-degrading genes in the hemocoel. However, we found that no significant difference in cuticle penetration was found between the WT strain and the strain *Coh1^OE^*. When inoculations were conducted by topical application and injection, the strain *Coh1^OE^* also had the same pathogenicity as the WT strain. Nevertheless, we found that overexpression of *Coh1* impaired the saprophytic growth, and the colony of strain *Coh1^OE^* partially collapsed and produced fewer conidia than the WT strain, suggesting that a proper expression pattern of *Coh1* is important for *M. robertsii* to saprophytically produce normal conidia for infection of insects. All these data were shown in Fig S3 and discussed in the discussion section in the revised manuscript.

*WT-COH2-FLAG* and *ΔCoh1-COH2-FLAG* were actually the strains with *Coh2* overexpressed in the WT strain and the mutant *ΔCoh1*, respectively. Since COH2 suppressed expression of genes for hemocoel colonization, it is assumed that overexpression of *Coh2* could further reduced the virulence of the mutant *Coh1*. However, via injection, the virulence of the strains *WT-COH2-FLAG* and *ΔCoh1-COH2-FLAG* was the same, and not significantly different from the mutants *ΔCoh1, ΔCoh2, and ΔCoh1::ΔCoh2*. These results further suggests that in addition to regulating hemocoel colonization with COH1, COH2 could have other uncharacterized functions. This is discussed in the discussion section.

**Comment 12:** Is there indeed a stronger melanization reaction and are antimicrobial peptide genes induced more strongly?

**Response:** According to this suggestion, we compared the immune responses of *Galleria mellonella* larvae to fungal infection by assaying the phenoloxidase (PO) activity and the expression of two antimicrobial peptide genes (gallerimycin and defensin). The tested fungal strains were the WT strain, the mutants *ΔCoh1*, *ΔCoh2*, and the *Coh2*-overexpressing strains *WT-COH2-FLAG* and *ΔCoh1-COH2-FLAG.*

The PO activity assay showed that fungal infection reduced the PO activity in the *Galleria mellonella* larvae. The insects infected by the WT strain, and the mutants *ΔCoh1*, *Coh2^OE^* and *ΔCoh1-Coh2^OE^* had the same PO activity, but they all showed lower PO activity than the insects infected by the mutant *ΔCoh2*. qRT-PCR analysis showed no significant difference in the expression levels of the gallerimycin and defensin gene between the insects infected by all tested strains. The new data were shown in Fig S2J, K, and discussed in the discussion section in the revised manuscript.

**Comment 13:** The generation of a Coh1-Coh2 double mutant and its phenotypic analysis after injection would allow determining whether the Coh1 phenotype is only mediated through COH2 degradation.

**Response:** According to this comment, we constructed the Coh1-Coh2 double deletion mutant *ΔCoh1׃׃ΔCoh2* (Fig. S1G). Since COH1 deactivates COH2-mediated repression of genes for hemocoel colonization, it is assumed that deleting *Coh2* in the *Coh1* mutant *ΔCoh1* could increase virulence of the mutant. However, via topical application and injection, the virulence of the double deletion mutant *ΔCoh1׃׃ΔCoh2* was not significant from the single deletion mutants *ΔCoh1* and *ΔCoh2*, but they all had significantly lower virulence than the WT strain. This could be due to the fact that Coh2 have other uncharacterized functions than repression of genes for hemocoel colonization. The new data were presented in Fig S9 and discussed in the dissection section in the revised manuscript.

**Comment 14:** Of note, the fungal burden should be measured in all in vivo experiments just prior to the onset of larval death, for both infection models.

**Response:** According to this suggestion, fungal burden was measured with qPCR in the dying insects that were infected with *M. robertsii* via topical application or injection. The new data were shown in Fig S2F and G in the revised manuscript. The data showed that the fungal burden in the insects infected by the WT strain was significantly higher than the mutants *ΔCoh1* and *ΔCoh2*, and the *Coh2*-overexpressing strains WT-*COH2-FLAG* and *ΔCoh1-COH2-FLAG*.

**Comment 15:** 3. The authors provide an interesting explanation for the lack of rescue of the Coh1 mutant phenotypes by the expression of the wild-type gene. However, they still cannot formally exclude that the Coh1 phenotype is due to a second-site mutation. The easiest to exclude this possibility would be to use the Coh1 overexpression construct using the *PTef* promoter. The alternative would be to knock-in back the wild-type gene in the deletion mutant.

**Response:** The “complemented strain”, constructed by putting the wild-type Coh1 gene back into the deletion mutant *ΔCoh1*, actually overexpressed the *Coh1* gene during the saprophytic growth (Fig. S1E). In the revised manuscript, we analyzed the phenotype of the “complemented strain”. Via injection of conidia into the hemocoel where Coh1 expressed in the WT strain, the “complemented” strain had the same virulence as the WT strain, which both showed higher virulence than the deletion mutant *ΔCoh1*. So, in terms of hemocoel colonization, we rescued the *Coh1* mutant phenotype in the “complemented strain”. However, we found that the saprophytic growth of the “complemented strain” was impaired with reduced conidial yield and abnormal colony phenotype on the PDA plates. According to the reviewer’s suggestion, we constructed the *Coh1-*overexpressing strain *Coh1^OE^* with *Coh1* driven by the constitutive promoter *pTef* to investigate whether the abnormal phenotype of the “complemented strain” resulted from the expression of *Coh1* during saprophytic growth. Similar to the “complemented strain”, the strain *Coh1^OE^* also showed reduced conidial yield and abnormal colony phenotype, but had the same virulence as the WT strain and the “complemented” strain when inoculation was conducted by direct injection of conidia into the hemocoel. All these data were included in the revised manuscript.

For *M. robertsii*, so far it has been not technically feasible to do knock in back a wild-type gene in its deletion mutant.

We would also like to mention to the reviewer that in the first submission we used two ways to exclude the possibility that the mutant *ΔCoh1* phenotype is due to a second mutation. First, Southern blotting analysis showed that there was one copy of the foreign phosphinothricin resistance gene *bar* in the three randomly-selected isolates (*ΔCoh1*#1, *ΔCoh1*#2, and *ΔCoh1*#3) (Fig S1C), suggesting that the insertion of the foreign DNA only resulted in deletion of *Coh1* based on homologous recombination and no ectopic integration occurred. Second, and no differences were observed between the three isolates (*ΔCoh1*#1, *ΔCoh1*#2, and *ΔCoh1*#3) in all assays.

**Comment 16:** With respect to the hypothesis of the Hat1 action being mediated through repression of Hdac1 expression, the authors should establish whether Hdac1 overexpression from the *pTef* promoter overrules the Hat1 deficiency phenotype as regards Coh1 expression.

**Response:** According to this suggestion, we constructed the strain *ΔHat1-Hdac1^OE^* with *Hdac1* overexpressed, driven by the promoter *pTef*, in the deletion mutant *ΔHat1*. qRT-PCR analysis showed that during hemocoel colonization the expression level of *Coh1* in the strain *ΔHat1-Hdac1^OE^* was lower than in the strain *ΔHat1* (Fig S4C in the revised manuscript), indicating that *Hdac1* overexpression did overrule the *Hat1* deficiency phenotype as regards *Coh1* expression.

**Comment 17:** 4. One important question remaining is how the expression of Hat1 gets decreased under conditions of growth in naive cell-free hemolymph. The authors should indicate whether the expression of this gene is affected when Coh1 or Coh2 are overexpressed or in the deletion mutant lines.

**Response:** As described above, the hemolymph used in this study contained hemocytes. We cannot obtain enough fungal biomass from the real hemocoel of the infected insects to prepare sufficient RNA for qRT-PCR analysis, so we compared *Hat1* expression between the WT strain and the mutants *ΔCoh1* and *ΔCoh2*, and *Coh1*-overexpressing strain *Coh1^OE^* and *Coh2*-overexpressing strain *WT-COH2-FLAG* in the hemolymph. The data showed that no significant difference in the expression of *Hat1* was found between these strains. The data was shown in Fig S11C in the revised manuscript. We also discuss the possible factors that trigged the reduction of *Hat1* expression during the hemocoel colonization in the discussion section.

**Comment 18:** The authors rely on constructs leading to the expression of tagged proteins. Their conclusions would be much stronger if they were to demonstrate that the resulting tagged proteins are functional, that is, that they can complement the corresponding deletion mutant phenotype (of course, this does not apply to constructs expressing truncated proteins.

**Response:** According to this suggestion, in the revised manuscript we investigated whether the fusion protein COH2׃׃FLAG had the same functions as the wild-type COH2 protein. To do this, we complemented the deletion mutant *ΔCoh2* with the construct where the fusion gene (*COH2׃׃FLAG*) was driven by *Coh2* promoter to result in the strain *ΔCoh2-PCoh2-COH2-FLAG*. Following assays showed the strain *ΔCoh2-PCoh2-COH2-FLAG* had the same virulence and saprophytic growth as the WT strain and the complemented strain *C-ΔCoh2*, suggesting that, COH2׃׃FLAG had the same function as the wild-type COH2 protein. The data were shown in Fig S7.

For COH1 fusion proteins, we cannot determine whether it had the same function as its wild-type version COH1 using the complementation method (as described above). This is because the deletion mutant *ΔCoh1* cannot be complemented with the wild-type *Coh1* gene, as described in our response to comment #15. However, the colony phenotype of the *Coh1-*overexpressing strain *Coh1^OE^* was the same as *Coh1-Myc* that constitutively expressing the fusion protein COH1׃׃Myc, which provided an indirect evidence to support that COH1 and COH1׃׃Myc had the same functions. This data were shown in Fig. S2 in the revised manuscript.

**Comment 19:** 6. Could COH1 be linked to ubiquitinylation processes (not to be addressed experimentally in this study)?

**Response:** According to this comment, we assayed the impact of COH1 expression on the ubiquitination of the COH2 protein by comparing the ubiquitination level of the COH2::FLAG protein in the strain *WT-COH2-FLAG* (expressing COH2::FLAG in the WT strain) with *Coh1^OE^-COH2-FLAG* strain (expressing COH2::FLAG in the *Coh1*-overexpressing strain). After immunoprecipitation using mouse anti-FLAG antibody, the ubiquitination of COH2::FLAG was assayed with rabbit anti-Ubi antibody, and the data showed that the intensity of smear ubiquitination band was higher in *Coh1^OE^-COH2-FLAG* than in the *WT-COH2-FLAG*, suggesting that COH1 increased the ubiquitination level of COH2::FLAG. The new data were included in the Fig 3F in the revised manuscript.

Minor comments:
**Comment 20:** a) Line 32 : COH2 not COH

**Response:** Changed.

**Comment 21:** b) Line 65: is Ref. 8 that relevant here ?

**Response:** Changed.

**Comment 22:**c) Lines 63-69 are taken with some modifications from one of their previous publications (ref. 10), a practice the authors may want to avoid.

**Response:** changed.

**Comment 23:**d) Line 116 and others: it is irritating to have figure legends in the middle of the text.

**Response:** changed.

**Comment 24:**e) Lines 135-144: does the Coh1 sequence encode a signal peptide (presumably not), a NLS?

**Response:** There were no putative signal peptide and NLS in COH1. Information added.

**Comment 25:**f) Lines 270-271: the logic of the experiment remains obscure and citing a single (lonely?) study does not help understanding it.

**Response:** We did use conventional method, i.e. pulling down with COH1::FLAG antibody and GC-MS analysis, to identify the component interacting COH1, but it was unsuccessful. In addition to the cited reference in the first submission, other references (cited in the revised manuscript) also show that transcription factors mostly work in a combinatorial model, which means a transcription factor and its interacting components may show a correlative expression pattern when responding to a stimulus. So, we assumed that if there is a regulatory component interacting with COH1, its expression might change when C*oh1* is overexpressed. We were lucky we found that COH1 interacted with COH2 by analyzing the RNA-Seq data of the *Coh1*-overexpressing strain *Coh1^OE^*.

**Comment 26:** g) Line 404: the COH2-BM sequence given here does not correspond to the consensus nor to the sequence shown in figures. This reviewer did not really observe an inverse repeat in the consensus motif; would a single nucleotide spacer be enough for the binding motif of a dimer, e.g., would the two "repeats" be on the same side of the double-helix?

**Response:** Thank the reviewer for this comment. We are sorry we used a wrong phrase for this sequence (TGACTCA); it is not an inverse repeat, but a palindrome sequence: a string with a 5’ to 3’ sequence that is identical to the 5’ to 3’ sequence of the reverse complimentary strand. Many transcription factors that form homo-dimers bind to palindrome sequences in the promoter regions of their target genes.

According to the ChIP-Seq analysis and EMSA assays, some nucleotides in the palindrome sequence can be changed to other nucleotides, so the COH2-BM sequence (AATGAGTCTC) shown in the Line 404 (in the first submission; Line 433 in the revised manuscript) was not exactly the same as the sequence shown in the Figure. Some transcription factors reported in previously published literatures can bind to palindrome sequences with altered nucleotides in the core sequence. For example, the EmBP-1, a wheat DNA binding protein of the basic leucine zipper (bZIP) class of transcription factors implicated in the mechanisms of abscisic acid mediated gene activation, can bind sites with altered nucleotides in the palindrome sequence (Niu X, Guiltinan MJ, DNA binding specificity of the wheat bZIP protein EmBP-1, Nucleic Acids Res, 1994, 22:4969-4978.).

According to previously published literatures, the number of nucleotides in the spacer in the palindrome sequences could vary, depending on the types of transcription factors that recognize the sequences. For some characterized transcription factors, a single nucleotide spacer was enough (e. g. Mori K, Ogawa N, Yanagi H, Yura T. Palindrome with spacer of one nucleotide is characteristic of the cis-acting unfolded protein response element in *Saccharomyces cerevisiae*. J Biol Chem. 1998, 273:9912-9920).

In light of the information provided in our response to this comment, we revised the manuscript.

**Comment 29:** h) Fig. 2B, D: Are the SDY and hemolymph conditions each normalized to the wild-type? Would it not be better to normalize the expression to a single condition, e.g., SDY wild-type, so that the expression in SDY and hemolymph can really be compared?

**Response:** According to this suggestion, we normalized the expression to a single condition (SDY wild-type) in the revised manuscript.
