# Supplementary material for: A novel cascade allows Metarhizium robertsii to distinguish cuticle and hemocoel microenvironments during infection of insects
Source: PLoS Biol. 2021 Aug 4;19(8):e3001360. doi: 10.1371/journal.pbio.3001360 (PMC8366996; doi:10.1371/journal.pbio.3001360)
Supplement: Attachment — Submitted filename: Point-to-point response.docx [file pbio.3001360.s016.docx]

Dear editors,

Thank you and reviewers again for valuable comments on our paper. We have amended the manuscript as suggested. Our point-to-point responses to reviewer and editor comments are pasted below.

Best regards

Sincerely yours

Weiguo Fang

**Editor comments**

**Comment 1:** Thank you for sending the S1_Data file containing the data underlying all the graphs shown in the figures. We have spotted some mistakes in the Fig. S11 tab that should be amended: please add Fig. S11A data, as it seems to be missing, and relabel the data currently shown as S11B Fig – it should be S11C Fig.

**Response**: The data for Fig. S11A is added in the revised Data_S1. We also changed the label for Fig. S11B.

**Comment 2: P**lease also make sure you mention in the corresponding figure legends WHERE THE DATA CAN BE FOUND.

**Response:** Information added in the corresponding figure legends.

**Comment 3:** Please ensure that your Data Statement in the submission system accurately describes where your data can be found and also please make publicly available the RNA-Seq data that you have deposited in GenBank with accession number PRJNA637940.

**Response:** The RNA-Seq data is deposited in Genbank with accession number PRJNA637940, and is now publicly available.

**Comment 4:** *Blurb* Please also provide a blurb which (if accepted) will be included in our weekly and monthly Electronic Table of Contents, sent out to readers of PLOS Biology, and may be used to promote your article in social media. The blurb should be about 30-40 words long and is subject to editorial changes. It should, without exaggeration, entice people to read your manuscript. It should not be redundant with the title and should not contain acronyms or abbreviations. For examples, view our author guidelines: https://journals.plos.org/plosbiology/s/revising-your-manuscript#loc-blurb

**Response:** a blurb is submitted.

**Reviewers' comments**

Rev. 3:

The authors have to be lauded for their efforts to address the issues raised by reviewers.

**Comment 5:** A critical point is that of the validation of their model in vivo during infection. To this end, the authors have used several tagged constructs to monitor whether the genes under investigation are indeed expressed in vivo. They rely on fluorescence to assess reporter expression. While in some cases such as Fig. 1E, the results are clear-cut, they are less so in other instances such as Fig. 3D and especially Fig. 5G and Fig. S11E. The fluorescence should be measured on several microscopic fields and rigorously quantified, subtracting the general background of the picture.

**Response:** According to this suggestion, using the Image J software, the background of the pictures was subtracted, and the fluorescence intensity of the fungal cells was then quantified. The detailed description of the method and the obtained new data were included in the revised manuscript. A new literature was cited.

The figures shown are actually representatives of multiple microscopic fields, and all photos were taken with the same exposure time. In figures Fig.5G, Fig. S11E, we used new pictures that had better quality with better background.

**Comment 6:** The results displayed in Fig. S8C showing a nuclear localization of COH2 occurring even when Coh-1 is overexpressed is puzzling since the model of the authors is that COH1 gets expressed only when the fungus is in the hemolymph and leads to the degradation of COH2. In addition, since COH1 lacks a nuclear localization signal, it may not reside in the same subcellular compartment, raising the possibility that COH2 in the nucleus might be "immune" from the action of COH1 in the cytoplasm. Presumably, these data have been gained with the fungus grown on SDY medium (the conditions are not described in the text nor in the figure legend). Would it be possible to use this strain in infections, even though the functionality of the N-GFP-COH2 fusion has not been tested? Can the authors exclude the possibility that adding GFP to the N-terminal side prevents COH1 from binding to COH2?

**Response:** Thank you very much for this comment. According to this suggestion, we constructed a new strain *COH1-HA/COH2-N-GFP* simultaneously expressing the proteins COH1::HA and COH2-N::GFP, with which we did new Co-IP experiments to assay the interaction between COH1::HA and COH2-N::GFP. The results showed that COH1::HA did not physically contact COH2-N::GFP (Fig. S8D in the revised version). However, as shown in the Fig. S6C, the protein COH1::HA physically interacted with the COH2-N::Myc. Therefore, adding GFP to the N-terminal side prevents COH1 from binding to COH2-N. In addition, the protein COH2-N::GFP did not undergo dimerization (Fig. S5C). Taken together, the protein COH2-N::GFP is not suitable for assaying the impact of COH1 on the entry of COH2 into the nucleus. Since construction of fusion protein COH2::GFP was not successful, we were unable to further assay the impact of COH1 on the entry of COH2 into the nucleus. Nevertheless, this does not hurt the conclusion that the interaction between COH1 and COH2 induces the degradation of the COH2::FLAG protein by the proteasome pathway.

All new data about the experiment with the protein COH2-N::GFP was still presented in the Figure S8 in the second revision.

In the Fig. S8C in the first revision, the data were gained in the SDY medium (this information added in the figure legend in the second revision). As suggested by the reviewer, we also assayed the localization of COH2-N::GFP during the real hemocoel colonization, and the results showed the COH2-N::GFP was also localized in the nuclei. This new data was also included in Fig. S8C in the second version.

**Comment 7:** The authors should also mention in the Discussion another consequence from the analysis of the double Coh1-Coh2 mutant. One might have reasoned that the Coh1 deletion phenotype might be solely caused by the ectopic stabilization of COH2. That the double mutant is still less virulent strongly suggests that COH1 is regulating other target proteins, in line with the results of the RNAseq analysis.

**Response:** Discussion added according to this suggestion.

**Comment 8:**

The clarity of the manuscript could be substantially improved by clearly stating the conditions used: the authors repeatedly refer to "hemocoel colonization" when they are using the assay of incubating the fungus in collected hemolymph that has been diluted with an anticoagulant solution (lines, 103, 120, 137, 225, 227, 248, 268, 272, 289, 385, 463, 468, 488, 506, 556, 559, 560, 620?, 631, 637, 788950, 961, 1035, 1425 and likely others I missed. It is important to use a term such as "surrogate hemocoel colonization" to make the distinction between artificial conditions and the in vivo situation. The use of the term "real hemocoel" when referring to in vivo conditions does not suffice. By the same token, the term "cuticle penetration" should likewise be avoided (lines 532, 554, 558).

**Response:** According to the reviewer’s suggestion, the terms “surrogate hemocoel colonization” and “real hemocoel colonization were used where appropriate in the second revision

In this study, two conditions were used to show cuticle penetration. One is the real cuticle penetration by growing the fungus on the real insect cuticle, and we still used cuticle penetration for this condition. The other one is to grow the fungus in the cuticle medium using the insect cuticle (grounded in to small pieces) as sole carbon and nitrogen source, and in the second revision we did not use cuticle penetration for this condition, and instead specified the condition by stating “growth in the cuticle medium”.

Minor points:

**Comment 9:** a) it might be desirable to use scatter plots when displaying the results of gene expression measured by RTqPCR.

**Response:** We used scatter plots to display results about gene expression measured by RTqPCR. But for three figures (Fig. S4B, Fig. S11D and Fig. S11G) that showed the results about many genes, we think scatter plots could be too busy, so histograms were still used in these three figures.

**Comment 10:** b) Line 318: please, change "COH1 physically interacted with" to "COH1 can physically interact with": these are artificial conditions in which the proteins are overexpressed. Whether the two proteins interact in vivo remains a possibility, but see also above.

**Response:** Changed.

**Comment 11:** c) Fig. S2F-G: the Y-axis caption should be changed as qPCR was used.

**Response:** Changed.
